# Cost-effectiveness of an integrated lifestyle intervention for obesity in southern Italy: a model-based simulation of costs, health outcomes and gastrointestinal cancer burden

**DOI:** 10.3389/fpubh.2026.1956296

**Published:** 2026-10-29

**Authors:** Maurizio Polignano, Davide Guido, Ornella Rotolo, Antonella Bianco, Giuseppe Dalfino, Michelangelo Armenise, Gianluigi Giannelli

**Affiliations:** 1 Scientific Direction, National Institute of Gastroenterology IRCCS “Saverio de Bellis”, Castellana Grotte, Bari, Italy; 2 Unit of Data Science, National Institute of Gastroenterology IRCCS “Saverio de Bellis”, Castellana Grotte, Bari, Italy; 3 Clinical Trial Unit, National Institute of Gastroenterology IRCCS “Saverio de Bellis”, Castellana Grotte, Bari, Italy; 4 Laboratory of Movement and Wellness, National Institute of Gastroenterology IRCCS “Saverio de Bellis”, Castellana Grotte, Bari, Italy

**Keywords:** cost-effectiveness analysis, gastrointestinal burden, health policy, lifestyle intervention, obesity, public health intervention, simulation study

## Abstract

We assessed the cost-effectiveness of a publicly funded lifestyle programme (supervised exercise and nutritional counselling) for adults with obesity (BMI 30.0–34.9 kg/m^2^) in Southern Italy and its projected effect on colorectal and hepatocellular cancer burden. A deterministic cohort state-transition (Markov-type) model with annual cycles simulated 19,000 treatment episodes delivered to 16,736 adults over 20 years, compared with standard of care from societal and payer perspectives. Costs (2024 euros) and QALYs were discounted at 3%. Uncertainty was assessed by one-way and break-even analyses, a 10,000-iteration probabilistic analysis, and an extreme scenario compounding reduced efficacy, higher relapse and a 6% discount rate. The programme yielded 1,354.50 incremental QALYs (0.081 per treated adult) at a net societal cost of €30.06 million: ICER €22,192/QALY societal and €25,987/QALY payer, net monetary benefit €10.58 million at €30,000/QALY. Assuming that sustained weight loss reduced 50% of the excess cancer risk associated with obesity, the model projected a 1.44% relative reduction in incidence (7.2 cases avoided, 95% central range 2.1–16.3, €123,000 in avoided cancer care). The intervention was cost-effective in 67.7% of draws; under the extreme scenario the ICER rose to €183,641/QALY (probability <0.1%). Cost-effectiveness is conditional on sustained adherence and contained relapse rather than inherent.

## 1 Introduction

Obesity is a growing public health challenge in Italy, with particularly high prevalence rates in the southern regions. National surveys estimate that more than 10% of Italian adults are obese, and in Apulia the figure rises to 11.8%, amounting to nearly 465,000 individuals, well above the national average (1–5). Obesity is a major risk factor for a wide spectrum of gastrointestinal (GI) and metabolic diseases, including metabolic dysfunction-associated steatotic liver disease (MASLD), metabolic dysfunction-associated steatohepatitis (MASH), cirrhosis, hepatocellular carcinoma (HCC), and colorectal and pancreatic cancers, thereby placing a substantial burden on gastroenterology services and public health systems (6–11). This translates into high clinical, economic, and social costs, driven by the management of obesity-related comorbidities and premature mortality (12).

Despite this burden, most obesity interventions in Southern Europe remain fragmented, underfunded, and rarely evaluated using health economic methods. Traditional strategies, such as generic lifestyle advice or isolated dietary/physical activity programs, show limited long-term adherence and effectiveness (13–15). Comprehensive economic evaluations of multicomponent lifestyle interventions are scarce in the Italian setting, and most available studies focused on single modalities, often neglecting real world challenges such as patient dropout, sustainability, and the long-term cost-offsets generated by cancer prevention (16, 17).

National and European policy documents increasingly highlight the need for structured evaluations of preventive programs, to inform regional health planning and resource allocation (18, 19). Italian Health Technology Assessment (HTA) guidelines at national and regional levels (e.g., Agenas) further emphasize the need for transparent modeling and local adaptability (20, 21). In this context, we evaluated the cost-effectiveness of a fully publicly funded integrated programme combining supervised physical activity and structured nutrition counseling for adults with obesity. Using a simulation model, we aimed to estimate the clinical benefits, economic impact, and long-term sustainability of this intervention from both payer and societal perspective. To our knowledge, this is the first comprehensive economic evaluation of such a multicomponent programme in southern Europe.

## 2 Methods

### 2.1 Epidemiological study design

The model represents adults resident in the Apulia Region with class I obesity (BMI 30.0–34.9 kg/m^2^) and no current indication for bariatric surgery. Applying regional prevalence estimates (3, 4) yielded approximately 38,000 eligible individuals, of whom 50% were assumed to enroll: the programme is modelled as 19,000 funded treatment episodes delivered in annual tranches of 950 over the 20-year horizon. Because capacity is reallocated to patients returning after failure or relapse once the treatment-naive queue is exhausted, these episodes correspond to 16,736 unique adults and 2,264 re-treatments. Patients are exchangeable within health states and are not stratified by age or sex; all-cause mortality and baseline gastrointestinal cancer incidence were applied as crude national rates, so the cohort implicitly shares the age and sex composition of the reference population. Since gastrointestinal cancer incidence rises steeply with age, the direction of the resulting bias cannot be determined without individual-level enrolment data.

### 2.2 Computational study design

The study compares a structured, multicomponent lifestyle intervention (supervised physical activity and nutritional counseling) against standard of care (unstructured advice) for adults with Class I obesity (BMI 30.0–34.9 kg/m^2^).

The analytical framework captures the long-term clinical trajectories by simulating patient transitions across defined health states (i.e., active obesity, weight-loss maintenance, surgical eligibility, and all-cause mortality). Furthermore, the core architecture was augmented with a dedicated gastrointestinal (GI) oncology module to quantify the downstream prevention of Colorectal Cancer (CRC) and Hepatocellular Carcinoma (HCC) linked to sustained weight loss.

The analysis was conducted both payer and societal perspectives, incorporating direct medical and indirect (productivity loss) costs. The primary outcome was the incremental cost-effectiveness ratio (ICER) expressed as euros per quality-adjusted life year (€/QALY) gained. Secondary outcomes included (i) pure oncological cost offsets, (ii) Net Monetary Benefit (NMB), and (iii) probabilistic acceptability curves. Model conceptualisation, design, and transparency adhere to the ISPOR-SMDM Modeling Good Research Practices (16, 22) and the CHEERS 2022 reporting guidelines (Supplementary material S1) (23).

### 2.3 Interventions and comparators

The simulated public health intervention consists of a multicomponent, structured lifestyle programme integrating supervised physical activity and progressive nutritional counseling. To reflect real-world clinical implementation and behavioral change theories, the intervention is delivered through two parallel, stepwise pathways designed to gradually reduce external supervision and foster patient autonomy over a 12-month period. The physical activity pathway transitions from an intensive initiation phase (supervised individual sessions, 3 months) to a consolidation phase (group sessions, 3 months), concluding with an autonomous maintenance phase (free gym access with booster sessions, 6 months). Concurrently, the nutritional support is schematized into three progressive 4-month stages: an individualized start (one-on-one consultations and tailored dietary planning), a group support phase leveraging peer exchange, and a final educational maintenance phase focusing on long-term dietary adherence.

The programme relies on a multidisciplinary team, including kinesiologists, dietitians and primary care physicians, applying structured behavioral and motivational strategies to mitigate patient dropout. The design is modeled upon validated international best practices and scalable regional adaptations, such as the ‘Palestre della Salute’ (Health Gyms) framework in Italy, ensuring reproducibility across different publicly funded healthcare systems (24, 25). A detailed programmatic pathway is provided in Supplementary material S2.

The primary comparator (Standard of Care, SoC) is defined as unstructured, episodic lifestyle advice provided during routine primary or specialist care consultations. This standard pathway is characterized by minimal multidisciplinary support, high natural attrition rates, and limited long-term clinical efficacy (12, 26–29). This “no intervention” comparator represents natural disease progression: the probability of clinically significant weight loss was set to zero and no programme or project-management costs were accrued. Costs of routine, unstructured advice during ordinary consultations were assumed equal in both arms and therefore cancel in the incremental analysis. All other streams (direct medical, indirect and bariatric-surgery costs, and background mortality) accrue as in the intervention arm.

The choice of standard unstructured care as the primary comparator is guided by current clinical reality and aligns with both European and Italian HTA recommendations for evaluating population-level lifestyle interventions (20, 30). We chose not to evaluate pharmacological treatments (e.g., GLP-1 receptor agonists) or bariatric surgery as primary comparators. This decision avoids artificial cost-effectiveness comparisons between preventive lifestyle pathways and invasive or lifelong pharmacological escalation, acknowledging that structured lifestyle programs are universally required as the foundational tier of obesity management, regardless of subsequent medical or surgical interventions.

### 2.4 Model overview

#### 2.4.1 Computational framework and health economic settings

To assess the long-term clinical and economic impact of the multidisciplinary lifestyle intervention versus standard of care (unstructured advice), we developed a deterministic, aggregate-level (cohort) state-transition model of the Markov type, run in annual cycles with a half-cycle correction (31). The model is time-inhomogeneous rather than stationary: relapse is not applied in a patient’s year of enrolment, and benefit decay is activated only beyond the benefit-duration window. It is not an individual-level microsimulation; patients are exchangeable within states and the model carries no individual history. The mutually exclusive health states are active obesity (untreated or awaiting treatment), maintained weight loss, primary treatment failure, late relapse, programme drop-out, post-bariatric state and death (see Figure 1).

**Figure 1:**
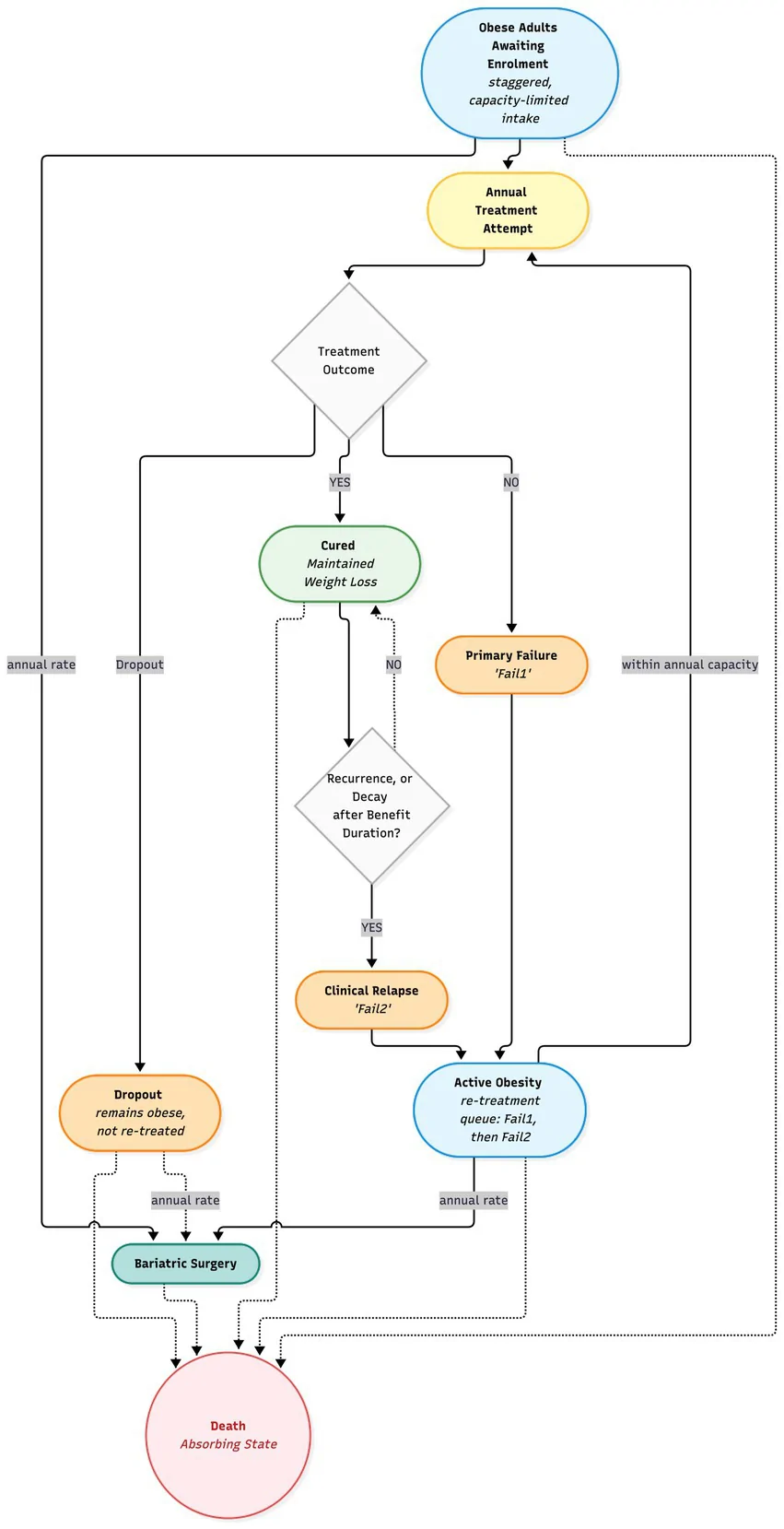
Flowchart of patient health states and transitions in the deterministic cohort model. Patients progress annually across mutually exclusive health states (active obesity, with clinical response, recurrence, bariatric surgery, death). Dropouts return to active obesity. Transition rates are literature-based.

Unlike traditional static obesity models, our architecture is built upon two interconnected engines: a core state-transition deterministic cohort model tracking metabolic trajectories, and a dedicated Gastrointestinal (GI) Oncology module. This modular design allows the simulation to link actively maintained weight loss to the downstream prevention of major malignancies.

The simulation runs on annual cycles over a 20-year time horizon. Analyses were conducted from both payer and societal perspectives, incorporating direct medical costs (e.g., outpatient visits, hospitalizations) and indirect costs (productivity losses) associated with active obesity. All monetary values are expressed in euros, updated to 2024; no inflation adjustment was applied, so that costs and quality-adjusted life years (QALYs) are discounted at the same real rate of 3% annually over the entire horizon, in accordance with Italian and European health economic guidelines and with CHEERS 2022 (21, 23). Italy has no statutory cost-effectiveness threshold. The primary willingness-to-pay (WTP) threshold was therefore set *a priori* at €30,000/QALY, a value within the range conventionally applied in Italian economic evaluations, with a stricter €20,000/QALY applied to the Extreme Scenario (17, 32).

Net monetary benefit is computed as NMB=(WTP×ΔQALY)−ΔCost , with both terms discounted at 3% per year.

Programmatic management (i.e., project management and administration) was modelled as a fixed cost envelope rather than a running percentage of programme costs. A total overhead budget (€2,776,000 in the Standard Scenario; €3,800,000 in the Extreme Scenario) was distributed evenly across the 20 project years and, like all other monetary streams, subject to the model’s discounting. The magnitude of this envelope is consistent with international evidence showing administrative expenditures ranging from 3.8–4.8% in efficient European single-payer systems to over 15% in multi-payer systems (33–35). All unit baseline costs were treated as constants within the model. The following flowchart (see Figure 2) illustrates the logical flow diagram of the model, from the initial input of clinical and macroeconomic parameters to the final aggregation of cost-effectiveness outcomes.

**Figure 2:**
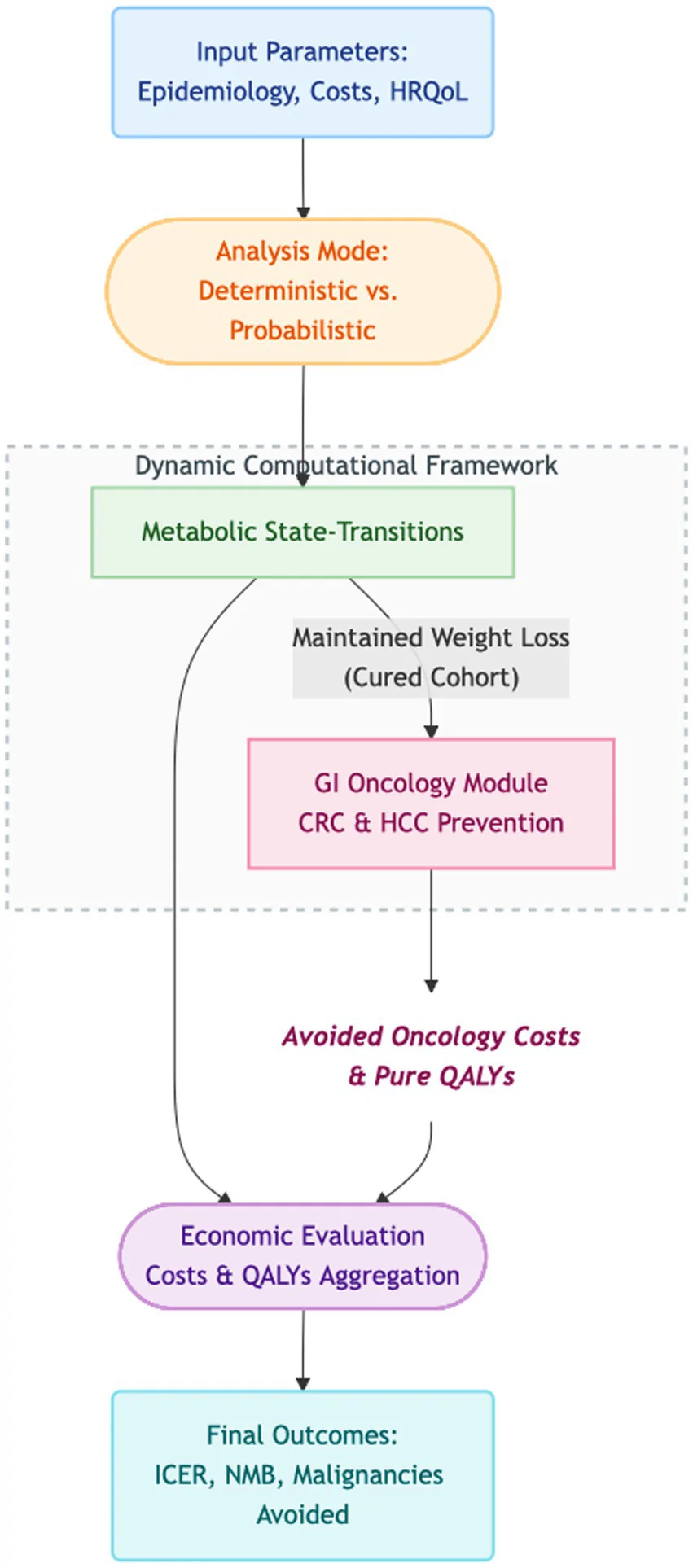
Logical architecture of the dynamic computational framework. The flowchart illustrates the integration between the core metabolic model and the GI Oncology module, demonstrating how sustained weight loss (“Cured Cohort”) actively drives the calculation of prevented malignancies and downstream oncological cost-offsets. CRC, colorectal cancer; GI, gastrointestinal; HCC, hepatocellular carcinoma; HRQoL, health-related quality of life; ICER, incremental cost-effectiveness ratio; NMB, net monetary benefit; QALY, quality-adjusted life year.

#### 2.4.2 Health states and transitions

Patients enter the model with active obesity and progress annually across mutually exclusive health states (see Figure 1). To ensure transparency, annual transition rates are governed by a specific set of literature-derived parameters:

- Probability of achieving a clinically significant weight loss (≥10% of baseline body weight) following the active intervention.
- Annual rate of weight regain or clinical relapse among previously successful patients, returning them to the active obesity state.
- Probability of programmatic attrition during the 12-month programme. Drop-out is modelled as a single event at the point of treatment and as a subset of the non-response budget (1 − p_success): it determines how non-responders are partitioned between a re-treatable pool (primary failure) and a non-re-treatable pool (drop-out), rather than the number of responders. Continuing loss of benefit after the programme is carried by relapse and decay. Newly cured patients are exposed to relapse in their year of entry, which is conservative.
- Probability of progression to bariatric surgery eligibility due to refractory severe obesity.
- All-cause background mortality rate, adjusted for obesity status.

Treatment durability is constrained by a benefit-duration window: beyond the fourth programme year an additional annual decay of 1% is applied to the maintained weight-loss state, in addition to relapse. The window is defined on the programme clock rather than on time since each patient’s own treatment, so that from year 5 onward it applies to the whole maintained weight-loss stock. Full set of transition rates, monetary inputs, utilities, bounds for sensitivity analyses, and references are reported in Section 2.5 and Supplementary material S3.

#### 2.4.3 Cohort tracing over time

To visualize population dynamics and state occupancy over time, cohort traces were generated across the 20-year simulation horizon (*N* = 19,000). Annual progression across mutually exclusive health states, encompassing untreated/waiting active obesity, maintained weight loss, primary treatment failure, clinical relapse, attrition, cumulative bariatric surgery, and all-cause mortality was tracked starting at baseline (*t* = 0) and plotted as a cumulative stacked area diagram.

Quality-adjusted life years and health-state costs were computed using a half-cycle correction: state occupancy was averaged between the beginning and the end of each annual cycle, so that transitions are valued at mid-cycle rather than at the cycle boundary. Event costs (i.e., programme delivery and bariatric procedures) accrue at the start of the cycle in which they occur. The correction only smooths the timing of transitions within each year: it does not reproduce sub-annual dynamics, and the annual cycle is aligned with the 12-month duration of the programme and with the annual basis on which all transition parameters are published. Cohort mass balance was verified at every cycle, with the sum of health-state occupancies plus cumulative deaths equaling the enrolled population to within a relative deviation of 3.8 × 10^−16^, the floating-point rounding limit of the arithmetic.

#### 2.4.4 GI-oncology module and parameterization

To quantify the oncological benefits of weight loss, we augmented the core state-transition structure with a dedicated Probabilistic Gastrointestinal (GI) Oncology Module.

This module dynamically links the actively maintained weight loss (the “Cured/Maintained” health state) to the reduction in relative risk (RR) for developing colorectal cancer (CRC) and hepatocellular carcinoma (HCC). Here, RR is defined as the risk ratio of a specific cancer outcome (CRC or HCC) conditional on the exposure factor, represented by the patient’s obesity status. Specifically, the model defines three exposure states: normal-weight individuals serve as the unexposed baseline reference (RR = 1.0); patients with active obesity represent the fully exposed group (RR_obese_); and patients who achieve maintained weight loss transition to a modified-exposure state (RR_treated_), reflecting a partial reversal of the adiposity-attributable excess risk. The computational engine then iteratively computes the annual number of major GI cancer cases avoided by multiplying the number of active patients in the maintained weight-loss state by the reduction in the relative risk (ΔRR = RR_obese_–RR_treated_) and the baseline cancer incidence.

Specifically, the module architecture incorporates baseline cancer incidence rates derived from Italian National registries (36), relative risks (RR) strongly modulated by active weight status (37, 38), acute Year-1 oncological management costs, chronic long-term follow-up costs (39), and acute (Year-1) and follow-up utility decrements (40). To reflect the biological reality that carcinogenesis unfolds over time, the GI-Oncology module incorporates a fixed 3-year latency period between entry into the maintained weight-loss state and the accrual of the associated reduction in relative risk (RR) for incident CRC and HCC. When a case is avoided in a given year, the associated cost and utility consequences are not attributed to that year alone but distributed over a five-year window from the year of avoidance, with the follow-up component attenuated year on year, giving a cumulative follow-up multiplier of 0.30 rather than the full 5 years. This mapping is applied downstream of the state-transition model, so that oncological outcomes depend on when weight loss was achieved without requiring tunnel states in the chain itself.

These avoided events are translated into downstream oncological cost offsets and pure QALY gains, isolating cancer prevention value (detailed in Supplementary material S3).

### 2.5 Costs, utilities, and model inputs

The parameters informing the economic model were derived from a comprehensive literature review, encompassing real-world observational studies, randomized controlled trials (RCTs), and established national health economic guidelines. To account for structural uncertainty and test the model’s robustness, two distinct Scenarios were parameterized: a Standard Scenario (reflecting the most plausible real-world outcomes) and an Extreme Scenario (representing highly conservative and pessimistic estimates). Bariatric surgery was modelled as an exogenous escalation pathway available to patients in the active-obesity state under both the intervention and the comparator, with identical transition rate, one-off cost and post-operative health-state utility in the two arms. The annual transition rate was derived from the 2024 SICOB national survey (41), which reports 1,725 bariatric procedures performed in Apulia applied to the regional population with obesity (approximately 465,000 residents, 12% of the Apulian population) this corresponds to approximately 0.37% per year. Because a proportion of intervention patients leave the active-obesity state through sustained weight loss, the comparator accrues more surgical procedures over the 20-year horizon (1,110 versus 1,003). This differential generates a modest cost offset in favour of the intervention but, since the post-bariatric state carries a utility premium equivalent to that of maintained weight loss (42), it simultaneously accrues QALYs to the comparator; the net contribution of the surgical channel to the incremental QALY total is therefore slightly negative (−41.0 QALYs in the Standard Scenario).

#### 2.5.1 Clinical efficacy and programmatic attrition

The success rate of the non-surgical intervention (p_success) was defined as the proportion of patients achieving a clinically significant weight loss (≥10% of initial body weight), derived for the Standard Scenario from the intention-to-treat analysis of the real-world OPTIFAST 52-week programme (43) and, for the Extreme Scenario, from pessimistic trial limits (44, 45). Patient retention remains a critical barrier in conservative obesity management. The programmatic dropout rate (p_dropout) was derived from real-world cohort data (46) while the annual rate of clinical relapse (p_recurrence) was informed by observational outcomes (43) and, for the Extreme Scenario, by evidence on the loss of durability under aggressive dietary restriction (46). Background mortality rates and surgical transition rates were informed by epidemiological literature and national clinical guidelines (47, 48). Values for both Scenarios are reported in Table 1.

**Table 1:** Key structural parameters of the simulation model.

Parameter	Standard scenario	Extreme scenario (stress test)
Clinical efficacy and attrition		
Clinical success (≥10% weight loss)	64%	44%
Annual clinical relapse rate	23%	40%
Programmatic dropout rate	20%	30%
Benefit duration window	4 years	2 years
Annual benefit decay beyond window	1.0%	1.5%
Annual bariatric-surgery transition rate ᵃ	0.37%	0.37%
Annual all-cause mortality	1.0%	2.0%
Health-related quality of life		
Utility: active obesity	0.75	0.73
Utility: maintained weight loss	0.81	0.76
Utility: post-bariatric state ᵇ	0.81	0.76
Annual background utility decline	−0.01	−0.01
Cost inputs (2024 constant €)		
Direct medical cost, active obesity (€/month)	146	108
Indirect cost, active obesity (€/month)	75	50
Direct medical cost, weight-loss state (€/month)	110	86
Indirect cost, weight-loss state (€/month)	56	40
Bariatric procedure (one-off)	€8,500	€10,000
Programme cost per participant (12 months)	€3,000	€3,000
Programme management envelope (total)	€2,776,000	€3,800,000
Intervention micro-costing		
Cost for physical activity step 1^c^	€600/month	
Months^c^	3	
Cost for physical activity step 2^c^	€100/month	
Months^c^	3	
Cost for physical activity step 3^c^	€50/month	
Months^c^	6	
Cost for nutritional intervention step 1^c^	€70/month	
Months^c^	4	
Cost for nutritional intervention step 2^c^	€50/month	
Months^c^	4	
Cost for nutritional intervention step 3^c^	€30/month	
Months^c^	4	
Analytic settings		
Annual discount rate (costs and QALYs)	3.0%	6.0%
Willingness-to-pay threshold	€30,000/QALY	€20,000/QALY

The table compares clinical efficacy, health-related quality of life (utility) weights, and macroeconomic variables adopted for the standard scenario analysis and the extreme scenario. 𝐽erived from the SICOB 2024 national survey (1,725 procedures performed in Apulia) applied to the regional population with obesity (approximately 465,000 residents, 12% of the Apulian population); ᵇthe post-bariatric and maintained weight-loss states are assigned the same utility, as both represent sustained clinically significant weight reduction. ^c^Identical in both scenarios.

#### 2.5.2 Health-related quality of life (utilities)

Health-state utility values (Table 1) reflect a baseline utility of 0.75 for active obesity, with clinically significant, maintained weight loss yielding a utility premium of +0.06 in the Standard Scenario (+0.03 in the Extreme Scenario) (49, 50). This premium represents the difference in EQ-5D utility between the active-obesity and the reduced-weight health states, consistent with the state-transition structure of the model, rather than a gradient per unit of BMI (49). To simulate physiological aging over the 20-year time horizon, an annual background utility decline (−0.01) was applied uniformly across all health states, independent of weight-loss status (51).

#### 2.5.3 Health economics parameters

Direct and indirect healthcare costs associated with active obesity were extracted from national burden-of-disease models and dynamically reduced within the simulation upon patient transition to the “Cured/Maintained” state (52). Intervention micro-costing (encompassing the 12-month multidisciplinary physical and nutritional pathways) was calculated based on regional professional tariffs. Simulation parameters are summarized in Table 1. Complete parameter values, including monetary inputs, utilities, and bounds for sensitivity analyses, are detailed in Supplementary material S3.

#### 2.5.4 Oncological parameters (GI module)

To inform the dynamic GI-Oncology module, baseline cancer incidence rates were set at 82.5 cases per 100,000 inhabitants for colorectal cancer (CRC) and 21.4 cases per 100,000 for hepatocellular carcinoma (HCC) (36). The relative risk (RR) of developing these malignancies for patients with active obesity versus normal-weight individuals was assumed to be 1.30 for CRC (37) and 1.80 for HCC (38). Upon achieving clinically significant weight loss, the residual RR in the treated cohort was modeled at 1.15 for CRC and 1.40 for HCC, representing a 50% reversal of the acquired excess metabolic risk for both cancers (53). The treated-state relative risk is a derived quantity rather than an independent input: RR_treated_ = 1 + (RR_obese_ − 1) × (1 − effect_weightloss), bounded within [1, RR_obese_], so that no parameter draw can place a patient in maintained weight loss at lower risk than a normal-weight individual or at higher risk than an untreated patient with obesity. Because direct evidence on the reversibility of adiposity-attributable cancer risk after non-surgical weight loss is weak, the reversal fraction was not fixed at a point estimate but carried through the analysis as an uncertain parameter: bounded to [0, 1] by construction and sampled from a Beta distribution (mean 0.50, 95% interval ±0.05, capped at 0.80) in the probabilistic analysis.

Acute Year-1 oncological management costs were parameterized at €22,000 for CRC (39) and €25,000 for HCC (54, 55), followed by chronic annual follow-up costs of €3,700 and €5,000, respectively. The corresponding utility decrements applied were 0.28 (Year 1) and 0.15 (follow-up) for CRC (40) and a more severe 0.35 (Year 1) and 0.20 (follow-up) for HCC (56). Finally, a latency of 3 years was applied before the risk reduction materialized, reflecting the physiological lag between sustained weight loss and any measurable change in carcinogenesis, and preventing the overestimation of immediate oncological benefit in the early years of the simulation. The latency is a model assumption rather than an estimate and is applied identically to CRC and HCC.

### 2.6 Sensitivity analyses

To assess the robustness of the baseline results and address parameter uncertainty, we conducted deterministic, probabilistic, and Scenario-based sensitivity analyses in accordance with ISPOR guidelines (57).

#### 2.6.1 Deterministic sensitivity analysis (DSA)

One-way sensitivity analyses (OWSA) were performed to identify the structural drivers of the model. Each parameter was varied independently across a predefined range (95% confidence intervals or a ± 20% heuristic variation) and the effect on net monetary benefit was visualised in a tornado diagram. Health-state utilities were entered as the utility premium of maintained weight loss (u_treated − u_obese) with the absolute level held constant, and separately as the absolute level with the premium held constant, rather than independently: because incremental QALYs accrue as occupancy × (u_treated − u_obese), varying the two utilities independently would generate configurations in which maintained weight loss carries lower utility than active obesity, which the probabilistic analysis excludes by construction. Threshold (break-even) analyses were run on the most influential parameters.

#### 2.6.2 Probabilistic sensitivity analysis (PSA)

A second-order PSA was run over 10,000 Monte Carlo iterations. Transition rates (p_success, p_dropout, p_recurrence) and health-state utilities were drawn from Beta distributions, cost inputs from Gamma distributions, and the benefit-duration window from a discrete uniform distribution; baseline incidences and relative risks in the GI-Oncology module were drawn from log-normal distributions. The draws were plotted on the cost-effectiveness plane, and a cost-effectiveness acceptability curve (CEAC) was generated across a continuum of willingness-to-pay thresholds, with primary focus on €30,000/QALY.

Five parameter pairs were sampled hierarchically rather than independently. In each, the dependent parameter was derived from its parent through a sign-constrained, bounded increment, so that no draw could reverse the ordering the model assumes on clinical grounds: the maintained weight-loss and post-bariatric utilities as the active-obesity utility plus a utility premium (Gamma-distributed by method of moments, capped at twice its mean); the treated-cohort relative risk by applying a bounded reversal fraction to the obesity excess risk alone; and the weight-loss-state direct and indirect costs by subtracting from each a separately sampled non-negative differential. This excludes draws in which remission lowers utility, raises oncological risk or increases expenditure, while leaving the marginal distributions of the parent parameters unaltered.

### 2.7 Extreme scenario analysis

An Extreme Scenario was modelled to identify the economic breaking point of the intervention. It compounds reduced clinical efficacy, elevated relapse, a shortened benefit-duration window, higher background mortality and adverse macroeconomic conditions, and was evaluated against a constrained WTP threshold of €20,000/QALY. It is a deliberately compounded boundary test rather than a plausible projection. Although Italian and European HTA guidelines typically recommend discount rates between 0 and 5% (with a 3% Standard Scenario), a 6% rate was deliberately adopted for this Scenario. Because the analysis is conducted in constant euros, this represents a true 6% real discount rate applied uniformly to costs and QALYs. Because benefits accrue later in the horizon than programme costs, this choice penalizes the intervention, although in isolation its effect is modest (Section 3.5); it is intended as a pure economic stress test rather than as standard practice (all values in Table 1).

### 2.8 Technical verification and quality assurance

Before integration with empirical inputs, the computational framework was verified in an isolated environment through an automated suite of 84 unit and integration tests. These cover four domains: (i) the arithmetic integrity of the deterministic core, including behaviour under extreme boundary states; (ii) the stability and seed reproducibility of the probabilistic and gastrointestinal modules; (iii) the separation of payer and societal cost streams; (iv) and the structural coherence of the oncological module, verifying that the treated-cohort relative risk remains bounded within [1, RR_obese_] across all probabilistic draws. Full specifications and verification logs are provided in Supplementary material S4.

### 2.9 Model validation

Beyond the technical verification described in Section 2.8, the model was assessed against the validation domains recommended by the ISPOR-SMDM Task Force. Face validity was established through structured review by the multidisciplinary author team, which includes kinesiology, clinical nutrition and internal medicine expertise alongside health economics and data science, so the health-state structure, the cohort trace and every input parameter were examined for clinical and economic plausibility against regional practice before the economic results were generated. Face validity was therefore assessed internally; independent external review of the model structure was not undertaken. Cross-validity was assessed against published economic evaluations of multicomponent lifestyle programmes (see Discussion). External validity was assessed against an independent observational cohort from the same institution and regional setting in which a kinesiology-supervised exercise and dietary counselling programme for adults with MASLD yielded a mean within-programme gain of 0.081 QALYs per participant (95% CI 0.001–0.161) at an ICER of €17,778/QALY, with 55.6% of participants (95% CI 37.3–72.4) maintaining regular physical activity at 2-year follow-up (58). The relapse rate adopted here implies a two-year persistence of 59.3%, consistent with that observation. In the absence of external validation datasets with matching study designs, we contextualized our findings using the nearest available empirical data. While not directly comparable, given that observed gains reflect short-term, unadjusted outcomes, whereas our 20-year projections incorporate attrition, relapse, and discounting, the modelled estimates consistently represent the more conservative benchmark.

### 2.10 Software architecture and reproducibility

All economic evaluations, encompassing the deterministic Standard Scenario, the PSA, and the dynamic GI-Oncology module, were executed using a custom-built computational framework (Obesity Cost-Effectiveness Simulation Model v1.3.0). To ensure maximal transparency and align with open-science best practices, the mathematical engine and underlying source code are publicly available under an MIT License on GitHub.^1^

The software was developed in Python (v3.12) (59) using NumPy (v2.3.2) (60) and Pandas (v2.3.1) (61) for vectorized state-transition matrix operations, while Plotly (v6.2.0) (62) provided high-resolution graphical rendering. The model was deployed via a Streamlit (v1.51.0) (63) interactive web interface. This architecture embeds automated integrity checks natively within the code to prevent computational errors across the 20-year simulation horizon.

### 2.11 Ethics statement

No ethical approval was required as no primary data collection or individual patient information was involved.

## 3 Results

The simulated integrated lifestyle intervention for the 19,000-patient cohort generated substantial clinical and economic benefits compared to standard unstructured care over a 20-year time horizon. The distribution of the cohort across the mutually exclusive health states over the simulation period is depicted in the state-transition trace (see Figure 3).

**Figure 3:**
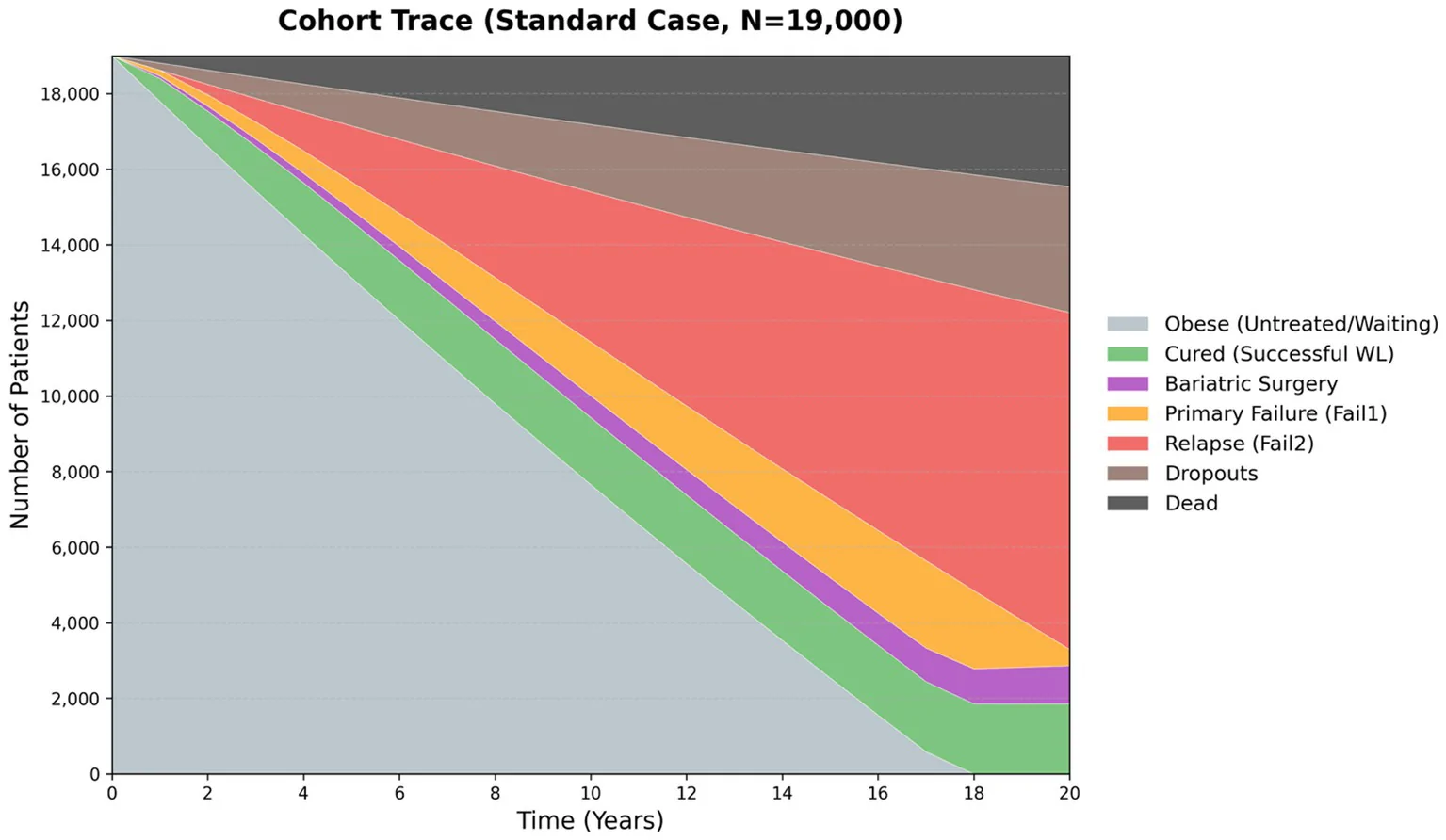
Standard Scenario. Cohort trace (*N* = 19,000) showing the distribution of patients across model health states over the 20-year time horizon.

### 3.1 Clinical outcomes and GI-oncology impact

The dynamic GI-Oncology module quantified the downstream oncological signal of sustained weight loss. The intervention yielded 1,352.71 incremental QALYs from the core metabolic model over the 20-year horizon, equivalent to 0.071 QALYs per funded treatment episode (19,000 episodes) or 0.081 QALYs per unique adult treated (16,736 adults, with 2,264 re-treatments). In patients maintaining clinically significant weight loss, the model reversed half of the excess adiposity-attributable risk for both malignancies (relative risk falling from 1.30 to 1.15 for CRC and from 1.80 to 1.40 for HCC). Tempered by real-world adherence and relapse across the full cohort, this mechanistic effect translated into a 1.44% relative reduction in the cumulative incidence of obesity-driven CRC and HCC over the horizon, equivalent to 7.21 avoided malignancies (4.26 CRC and 2.95 HCC). Because the module captures morbidity-related disutility only and excludes any survival benefit from avoided cancer-specific mortality, these averted events contributed a deliberately conservative 1.79 QALYs, bringing total incremental QALYs to 1,354.50. Excluding the oncological module altogether returns an ICER of €22,312/QALY against the integrated €22,192/QALY, so the contribution of the oncological extrapolation to the economic conclusion is visible to the reader.

### 3.2 Health economic outcomes and cost-effectiveness

The gross incremental cost of implementing the multidisciplinary programme, including centralized project management overhead, was substantial, but the prevention of downstream complications progressively offset this initial investment. Factoring in all direct and indirect cost offsets from obesity remission and prevented cancers, the net total incremental cost was €30.06 million from the societal perspective and €35.20 million from the payer perspective, the difference corresponding to €5.14 million in avoided productivity losses. The full decomposition of the incremental cost by cost stream and by perspective is reported in Table 2. The integrated incremental cost-effectiveness ratio was therefore €22,192 per QALY gained from the societal perspective and €25,987 per QALY from the payer perspective. At the predefined willingness-to-pay threshold of €30,000/QALY the intervention was cost-effective under both perspectives, generating a societal net monetary benefit of €10.58 million and a payer net monetary benefit of €5.44 million (see Table 3). Avoided GI malignancies alone generated €123,171 in oncological cost offsets, encompassing both acute management and chronic follow-up care.

**Table 2:** Decomposition of the 20-year incremental cost by cost stream and perspective.

Cost stream (discounted at 3%)	Intervention	Standard of care	Increment (Δ)
Lifestyle programme (19,000 treatment episodes)	43,672.8	—	+43,672.8
Project management and administrative overhead	2,126.9	—	+2,126.9
Bariatric surgery	7,349.3	8,087.0	−737.8
Direct medical costs, health states	453,285.1	463,024.6	−9,739.5
Healthcare subtotal (payer perspective)	**506,434.1**	**471,111.6**	**+35,322.5**
Avoided CRC and HCC care (oncological offsets)	—	—	−123.2
Total incremental cost, payer perspective			**+35,199.3**
Indirect costs (productivity losses)	232,663.8	237,804.1	−5,140.3
Total incremental cost, societal perspective			**+30,059.0**

Costs in thousands of 2024 euros, discounted at 3% annually; the corresponding year-by-year streams are reported in Table 4. The healthcare subtotal defines the payer perspective; adding avoided productivity losses gives the societal perspective, so the difference between the two perspectives is exactly the €5,140.3 thousand of avoided productivity loss. Oncological offsets comprise first-year management and follow-up care for the 7.21 avoided cases (CRC €68.7 thousand; HCC €54.5 thousand). CRC, colorectal cancer; HCC, hepatocellular carcinoma. Values in bold denote subtotals and totals.

**Table 3:** Comparative input parameters and clinical/economic outcomes: standard scenario versus extreme scenario.

Parameter/outcome	Standard scenario	Extreme scenario
Annual relapse rate	23%	40%
Incremental QALYs–Core metabolic model	1,352.71	186.13
Incremental QALYs–GI oncology module	1.79	0.62
Total incremental QALYs	**1,354.50**	**186.75**
Incremental QALYs per treatment episode	0.071	0.010
Bariatric procedures (intervention vs. comparator)	1,003 vs. 1,110	873 vs. 906
Incremental cost, core model (societal)	€30,182,173	€34,337,873
Oncological cost offsets	−€123,171	−€42,820
Total incremental cost (societal)	**€30,059,002**	**€34,295,053**
Avoided GI malignancies (CRC + HCC)	7.21 (4.26 + 2.95)	3.45 (2.04 + 1.41)
Relative reduction in GI cancer incidence	1.44%	0.76%
Integrated ICER–societal	**€22,192/QALY**	**€183,641/QALY**
Integrated ICER–payer	€25,987/QALY	€187,627/QALY
Net monetary benefit–societal	**+€10,576,068**	**−€30,560,038**
Net monetary benefit–payer	+€5,435,757	−€31,304,557
NMB at a common €30,000 threshold ᶜ	+€10,576,068	−€28,692,553
PSA–mean ΔQALY (95% central range)	1,391.4 (483.7–2,847.5)	191.5 (22.8–525.8)
PSA–mean ΔCost (95% central range)	€29.69 M (€18.32–38.04 M)	€34.14 M (€31.73–35.62 M)
PSA–median ICER	€23,061/QALY	€213,766/QALY
PSA–Pr (cost-effective)	67.7% @ €30,000	<0.1% @ €20,000
PSA–mean ΔCost, payer (95% central range)	€34.94 M (€26.56–40.83 M)	—
PSA–median ICER, payer	€27,225/QALY	—
PSA–Pr (cost-effective), payer	57.6% @ €30,000	—
Break-even values (ICER = €30,000/QALY)		
Utility premium, maintained weight loss	0.045	—
Annual relapse rate	28%	—
Clinical success rate	52%	—
Direct medical cost, active obesity	€107/month	—

All monetary outcomes are integrated (deterministic) results from the societal perspective (direct + indirect costs); ᵃ Scenario NMBs computed at their respective thresholds are not directly comparable; this row reports both at €30,000/QALY. Break-even values indicate the level at which the integrated ICER reaches €30,000/QALY, holding all other parameters at Standard Scenario values. GI, gastrointestinal; Pr, probability. —, not estimated. Values in bold denote the primary integrated outcomes from the societal perspective.

### 3.3 Budget impact

Scaling the programme to the Apulian population produced a manageable budget impact. Total 20-year expenditure was €739.1 million under the intervention against €708.9 million under standard care, a cumulative incremental cost of €30.2 million. The annual net burden was highest in the first year (+€2.77 million) and declined monotonically thereafter (+€1.01 million in year 20) as accumulating cost offsets from obesity remission progressively absorbed a larger share of programme expenditure, although they did not fully offset it within the horizon (Figure 4; Table 4).

**Figure 4:**
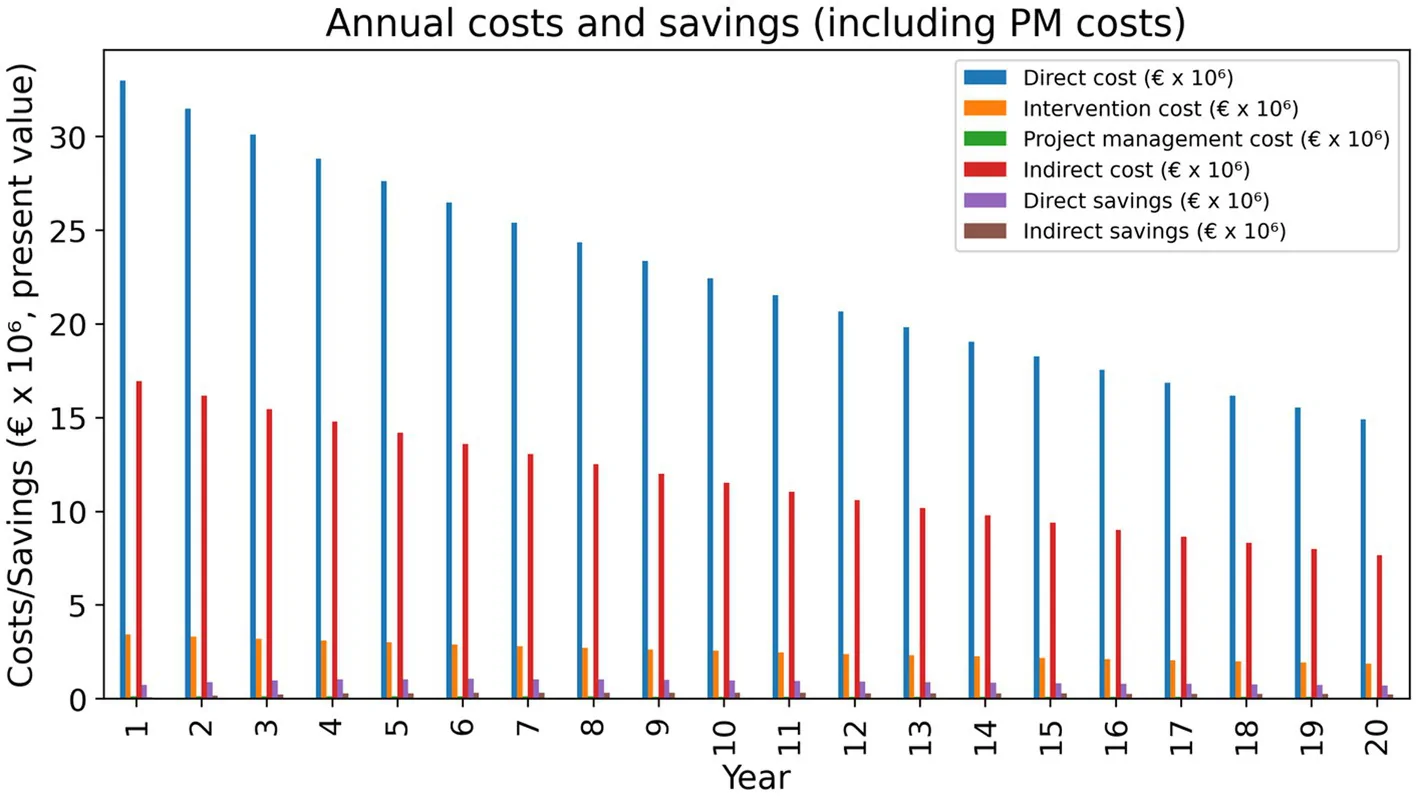
Annual net incremental budget impact (M€) over the 20-year simulation horizon. The column chart illustrates the yearly dynamic economic burden (investment minus dynamic savings) for the lifestyle intervention.

**Table 4:** Annualised cost components of the lifestyle intervention compared with standard care.

Year	Treatment episodes	Lifestyle programme	Bariatric surgery (intervention)	Project Mgmt.	Direct medical (intervention)	Indirect costs (intervention)	Total cost (intervention)	Direct medical (SoC)	Bariatric surgery (SoC)	Indirect costs (SoC)	Total cost (SoC)	Net incremental cost (Δ)
1	950	2,850.0	572.5	138.8	32,975.7	16,937.5	**53,474.4**	33,106.5	591.6	17,006.6	**50,704.7**	**+2,769.8**
5	950	2,532.2	456.2	123.3	27,611.8	14,174.9	**44,898.4**	28,153.2	497.5	14,460.7	**43,111.4**	**+1,787.0**
10	950	2,184.3	358.8	106.4	22,424.2	11,508.5	**36,582.2**	22,991.8	400.6	11,808.1	**35,200.4**	**+1,381.8**
15	950	1,884.2	285.7	91.8	18,273.7	9,376.6	**29,912.0**	18,777.7	322.6	9,642.6	**28,743.0**	**+1,169.1**
20	950	1,625.3	228.2	79.2	14,904.5	7,646.5	**24,483.7**	15,337.0	259.8	7,874.8	**23,471.6**	**+1,012.0**
Total (20 y)	19,000	43,672.8	7,349.3	2,126.9	453,285.1	232,663.8	**739,097.9**	463,024.6	8,087.0	237,804.1	**708,915.7**	**+30,182.2**

Costs in thousands of 2024 euros, discounted at 3% annually. Episodes: treatment courses initiated in each year (19,000 courses across 16,736 first-time participants and 2,264 reentries following primary failure or relapse). Programme: personnel, facilities and materials for the 12-month multidisciplinary pathway. Bariatric: one-off surgical costs, modelled identically in both arms; the comparator accrues more procedures (1,110 versus 1,003 over the horizon) because fewer of its patients exit the active-obesity state. PM, project management and administrative overhead. Direct med.: direct medical costs excluding bariatric procedures. Indirect: productivity losses. Displayed years are a sample of the 20-year horizon; the total row covers all 20 years. SoC, standard of care. Values in bold denote total annual costs per arm (no intervention and intervention) and the net incremental cost.

### 3.4 Sensitivity analyses

To evaluate the robustness of the Standard Scenario results against parameter uncertainty, both deterministic and probabilistic sensitivity analyses were conducted.

#### 3.4.1 Deterministic sensitivity analysis (DSA)

The one-way analysis identified the utility premium of maintained weight loss as the dominant driver of net monetary benefit, followed by the annual relapse rate (p_recurrence), the clinical success rate (p_success) and the direct medical cost of active obesity (cost_dir_obese). The absolute level of health-state utility is not itself a driver: holding the premium at 0.06 and shifting the level from 0.65 to 0.85 leaves incremental QALYs unchanged at 1,354.50 and the ICER at €22,192/QALY, because incremental QALYs accrue as occupancy × (u_treated − u_obese). Fluctuations in programmatic costs (e.g., dietetic or physical activity sessions) had a marginal impact on the overall cost-effectiveness (see Figure 5).

**Figure 5:**
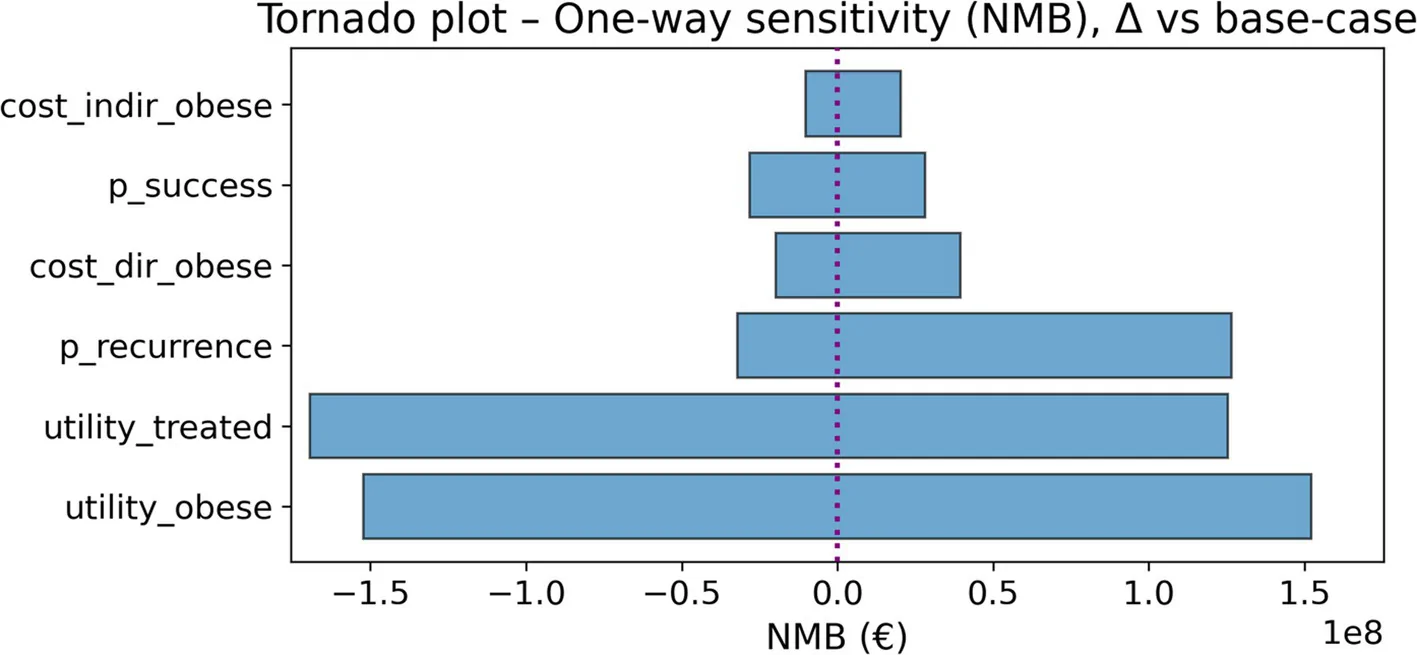
One-way sensitivity analysis of Net Monetary Benefit (NMB). Note: tornado diagram showing the effect of varying each parameter across its predefined range. Health-state utilities enter as the utility premium of maintained weight loss (u_treated − u_obese), with the absolute utility level varied separately at constant premium, so that no bar corresponds to a configuration in which maintained weight loss carries lower utility than active obesity. The vertical dashed line indicates the reference NMB value. Parameters are ordered by increasing width of effect, with the most influential at the bottom. utility_obese: health-state utility in active obesity; utility_treated: health-state utility in maintained weight loss; p_recurrence: annual rate of relapse; p_success: probability of achieving clinically significant weight loss; cost_dir_obese and cost_indir_obese: direct medical and indirect productivity costs in active obesity (€/month). Values on the horizontal axis are in units of €10^8^.

#### 3.4.2 Probabilistic sensitivity analysis (PSA)

The 10,000-iteration Probabilistic Sensitivity Analysis (PSA) mapped joint parameter uncertainty onto the cost-effectiveness plane (see Figure 6). The scatter plot illustrates the joint distribution of incremental costs and QALYs, with the 95% covariance ellipse enclosing the primary mass of Monte Carlo simulations and demonstrating the correlation between cost and effect estimates. From the societal perspective, the PSA yielded a mean incremental cost of €29.69 million (95% central range, €18.32–€38.04 M) and a mean QALY gain of 1,391.4 (95% central range, 483.7–2,847.5), with a median ICER of €23,061/QALY. All but one of the 10,000 draws fell in the north-east quadrant of the cost-effectiveness plane, that is more effective and more costly; a single draw was dominant, and none was dominated. The intervention was not cost-effective at €30,000/QALY in 32.3% of draws.

**Figure 6:**
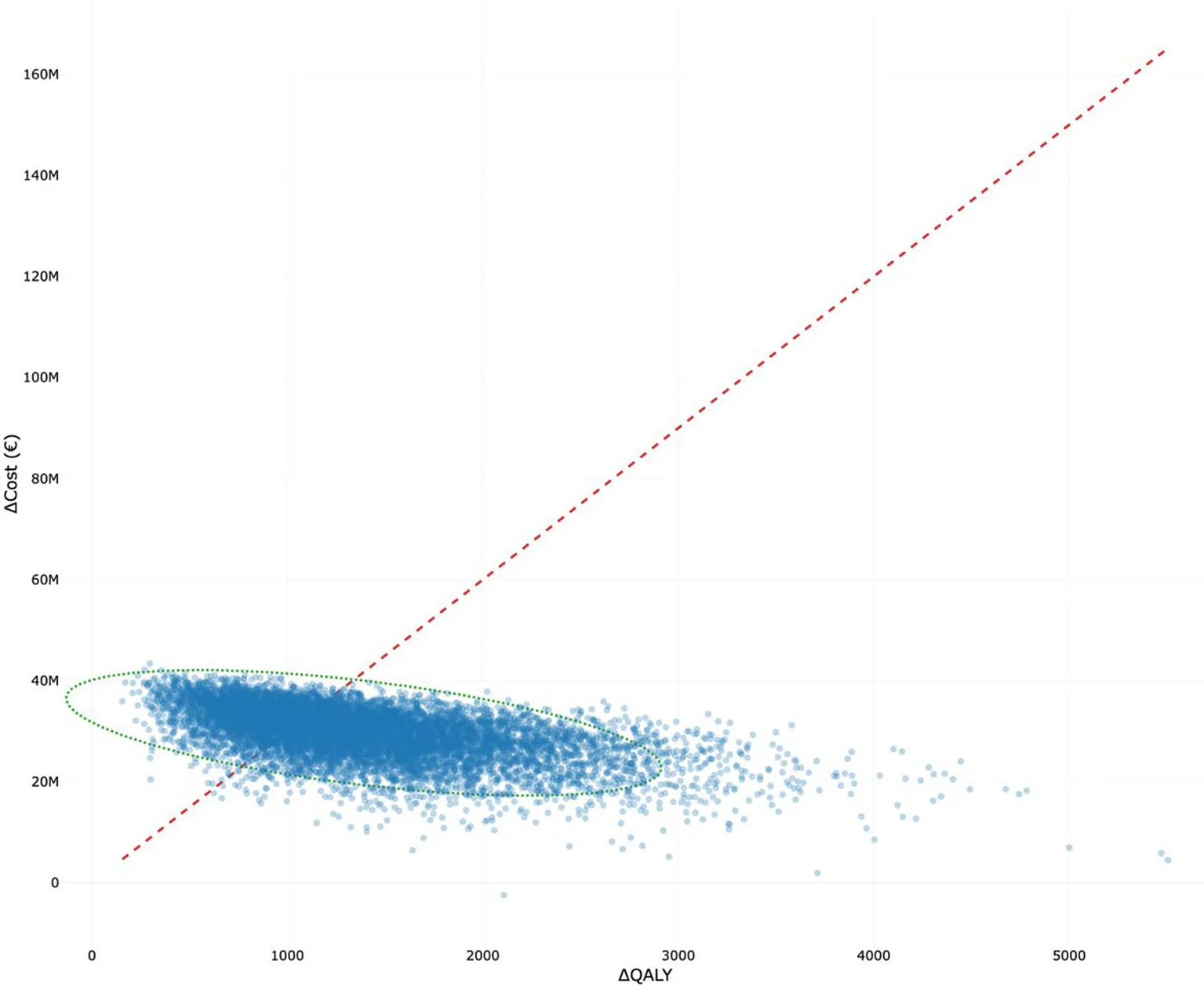
Cost-effectiveness plane of the intervention versus standard care in the standard Scenario (10,000 PSA iterations). A 95% covariance ellipse is shown.

The Cost-Effectiveness Acceptability Curve (see Figure 7) synthesizes decision uncertainty across willingness-to-pay (WTP) thresholds, indicating a 67.7% probability of cost-effectiveness at €30,000/QALY (57.6% from the payer perspective, with a median ICER of €27,225/QALY). Overall, cost-effectiveness remains favorable but contingent on parameter uncertainty, particularly clinical relapse and utility weights, while the oncological module contributed under 0.5% of incremental costs on average (0.41% in the Standard Scenario) and remained below 1% across most draws.

**Figure 7:**
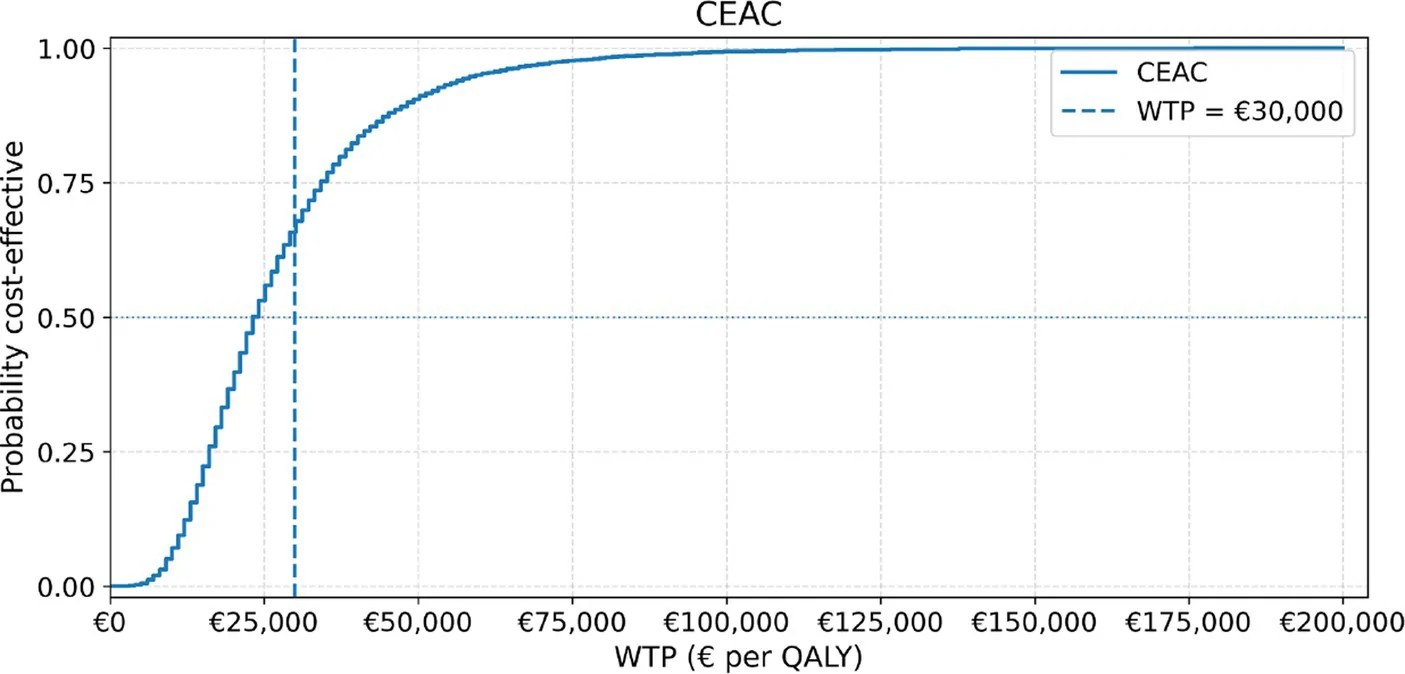
Cost-effectiveness acceptability curve (CEAC).

Complete outputs of the probabilistic sensitivity analyses, including parameter distributions and detailed cost-effectiveness metrics for both the Standard and Extreme Scenarios, are available in Supplementary material S5, S6, respectively.

### 3.5 Scenario analysis

To establish the economic breaking point of the intervention, a side-by-side comparison was conducted between the Standard and the Extreme Scenario (parameters in Table 1). Under stress-test conditions the deterministic analysis projected a marked reduction in QALY accrual (186.13 QALYs from the core model and 186.75 including the oncological module, roughly one seventh of the Standard Scenario total) together with a smaller number of avoided GI malignancies (3.45 cases, a 0.76% relative reduction). Net incremental costs were €34.30 million from the societal perspective and €35.04 million from the payer perspective, driving the integrated ICER to €183,641/QALY and €187,627/QALY respectively, roughly eight times the Standard Scenario value. Evaluated against the strict €20,000/QALY threshold, this yielded a net monetary benefit of −€30.56 million from the societal perspective (−€31.30 million from the payer perspective); at the €30,000/QALY threshold applied in the Standard Scenario the societal net monetary benefit remained strongly negative at −€28.69 million.” (The payer value −€31.30 million refers to the €20,000/QALY threshold; see Table 3).

The probabilistic analysis (10,000 draws) under Extreme Scenario parameters confirmed this shift, yielding a mean incremental cost of €34.14 million (95% central range, €31.73–€35.62 million) and a mean incremental QALY gain of 191.5 (95% central range, 22.8–525.8), with a median ICER of €213,766/QALY. The probability of the intervention being cost-effective at €20,000/QALY was below 0.1%.

The Standard Scenario conclusion is contingent on a utility premium of 0.06 for maintained weight loss: the integrated societal ICER reaches €30,000/QALY at a premium of 0.045 and rises to €44,325/QALY at 0.03. Equivalently, cost-effectiveness at that threshold requires an annual relapse rate below approximately 28% and a clinical success rate above approximately 52% (see Table 3). The gap between the two scenarios is produced by the compounding of three parameters acting on the QALY numerator rather than by the discount rate: applied individually to the Standard Scenario, the relapse rate (23 to 40%) raises the ICER to €52,368/QALY, the halved utility premium (0.06 to 0.03) to €44,325/QALY and the reduced success rate (64 to 44%) to €37,545/QALY, whereas the 6% discount rate alone raises it to €23,541/QALY.

### 3.6 Computational verification outcomes

The model passed all 84 predefined unit and integration tests with no deviation beyond floating-point tolerance, across the deterministic, stochastic, economic-synthesis and structural-coherence domains described in Section 2.8. Complete verification logs are provided in Supplementary material S4.

## 4 Discussion

These projections sit within the international evidence base, at its more conservative end. Our per-participant gain of 0.081 QALYs falls near the lower bound of the Look AHEAD range (+0.07 to +0.15 QALYs) (64) and below the DPP/DPPOS estimate (+0.14 QALYs) (65), while clearly exceeding lower-intensity European community programmes such as EuroFIT (66) (+0.01 QALYs at 5 years) and SITLESS (67), whose returns likewise proved contingent on effect maintenance. Two features of our design account for the difference from the North American trials: the cohort comprises adults with uncomplicated class I obesity rather than a prediabetic or diabetic population with greater scope for health gain, and enrolment is staggered across the full 20-year horizon, so that later entrants accrue benefit over a shorter residual period. That the ICER nonetheless falls below the €30,000/QALY threshold adopted here reflects the low unit cost of the programme relative to its sustained effect (64, 68).

The oncological dimension deserves particular attention, since obesity drives roughly 10% of colorectal and 19% of liver cancers in Italian men (69). Applying national incidence rates (36), the cohort was projected to develop approximately 499 major GI malignancies over two decades; by reversing half of the excess adiposity-attributable risk in patients who sustained weight loss, the programme was projected to avoid 7.2 of them, a 1.44% relative reduction, worth approximately €123,000 in offsets. This offset is immaterial to the headline ratio, and that is precisely the point: the module’s value lies not in large euro saving but in demonstrating that a non-pharmacological, population-scale programme is projected to bend the obesity-attributable GI-cancer incidence curve. This projection is a causal extrapolation from epidemiological association rather than an effect observed. Capturing this delayed benefit required mapping cost and utility kernels onto the specific year in which each avoided case accrues, which avoids the tunnel states normally needed to escape the memoryless propertythe gap between the reversal of half the excess risk assumed in maintainers and the diluted 1.44% realised across the cohort is governed by adherence, relapse and the three-year latency, the same constraint that governs the economic result.

We deliberately excluded anti-obesity pharmacotherapy, such as GLP-1 receptor agonists, from the comparator set. These agents are not universally reimbursed by the Italian NHS for uncomplicated obesity, and structured lifestyle programs remain the foundational tier of obesity management irrespective of subsequent escalation (27, 29). Supervised activity and nutritional counselling remain necessary even alongside pharmacotherapy, both to limit lean-mass loss during rapid weight reduction and to mitigate the pronounced regain observed after discontinuation. A cost-effective lifestyle pathway at approximately €22,000/QALY is therefore best understood as the structural precondition for any sustainable integration of pharmacotherapy into national health systems, rather than as its competitor.

Reimbursement status may nonetheless change within a 20-year horizon, and the direction of the resulting bias would fall on the comparator rather than on the intervention: were pharmacotherapy to enter standard care, the comparator would become both more effective and more costly, and the present analysis would then describe lifestyle care versus current unstructured care rather than versus pharmacological management. As an add-on, the arithmetic is dominated by acquisition cost: the modelled programme costs €3,000 per 12-month episode and is incurred once, whereas pharmacotherapy recurs annually at a multiple of that figure, so the cost-effectiveness of a combined pathway would depend on a future net price that cannot be sourced today. We therefore report this as a limitation rather than as a scenario.

The model implicitly assumes that participation and adherence are independent of socioeconomic position. Evidence on lifestyle interventions indicates otherwise: adherence tends to be higher among more advantaged groups, while obesity prevalence is inversely related to socioeconomic status (70). The programme could therefore prove cost-effective in aggregate while widening relative inequalities, an outcome a cohort-mean analysis cannot detect.

Several limitations temper these conclusions. The outcomes are model-based projections rather than empirical observations, and the efficacy parameters derive from international trials and real-world data in the absence of equivalent long-term Italian datasets. The source programmes also incorporated meal replacement, whereas the modelled intervention comprises supervised physical activity and nutritional counselling alone, so the base-case success rate is likely optimistic for this delivery model; the Extreme Scenario was constructed to stress-test exactly this transfer, reducing clinical success from 64 to 44%, halving the utility premium and nearly doubling relapse. The annual cycle length (with half-cycle correction) is a further simplification: it matches the 12-month duration of the programme and the annual basis of the published transition parameters, but it cannot represent sub-annual dynamics during the intervention year. We excluded other obesity-related cancers, such as breast, endometrial and renal, together with acute cardiovascular events, and the oncological QALYs capture morbidity-related disutility only, omitting the survival gains from avoided cancer-specific mortality; both the QALY gains and the savings reported here are consequently conservative with respect to these omitted outcomes. The GI module also applies a fixed three-year latency, which preserves computational tractability at the cost of a time-varying lag structure. Finally, the cohort trace over time shows an inflection at Year 18, when the treatment-naïve queue is exhausted and programme capacity begins to be absorbed by patients re-entering after relapse; while this geometry is an artifact of closed-cohort modelling, it mirrors the real-world transition to managing obesity as a chronic, relapsing condition.

Health-state utilities were drawn from a UK EQ-5D survey and applied to an Italian cohort. Because incremental QALYs depend strictly on the utility difference between active obesity and maintained weight loss rather than absolute baseline levels, re-anchoring to an Italian tariff leaves the results unchanged unless the premium itself shifts. The principal driver of uncertainty is therefore the utility premium itself; notably, its break-even value of 0.045 lies 25% below the base case, demonstrating resilience to geographical tariff variations.

Two further limitations concern the surgical pathway. The bariatric transition rate was derived from a national procedure count aggregated across all BMI classes and applied uniformly to a class I cohort, and the post-bariatric health state was assigned the same utility as maintained lifestyle-induced weight loss in the absence of Italian preference-based evidence distinguishing the two. Both assumptions were applied identically to the intervention and the comparator, so that the surgical channel does not systematically favour either arm.

Future evaluations utilizing open-population models, which allow for continuous patient enrollment rather than a bounded regional cohort, could smooth this mathematical artifact and provide a more stabilized projection of the long-term programmatic budget impact.

## 5 Conclusion

This simulation indicates that an integrated lifestyle intervention for obesity is cost-effective within standard thresholds and is projected to mitigate long-term gastrointestinal cancer risk over a 20-year horizon. However, these outcomes depend strictly on long-term behavioral persistence: economic viability requires maintaining an adequate quality-of-life premium and controlling clinical relapse, failing under compounding extreme stress-test scenarios. These findings support scaling structured lifestyle programs within public healthcare systems as a component of obesity management with a potential contribution to the primary prevention of gastrointestinal cancer. To manage long-term forecasting uncertainties, the model is deployed as a fully transparent, open-access web application, enabling local stakeholders to dynamically re-evaluate and recalibrate parameters to inform evidence-based health policies.

## Data Availability

The datasets presented in this study can be found in online repositories. The names of the repository/repositories and accession number(s) can be found in the article/Supplementary material.

## Footnotes

1
https://github.com/Pharma-Py/Obesity_treatment_calculator

## Glossary

AIFA: Agenzia Italiana del Farmaco (Italian Medicines Agency)
BMI: Body Mass Index
CEAC: Cost-Effectiveness Acceptability Curve
CHEERS: Consolidated Health Economic Evaluation Reporting Standards
CRC: Colorectal Cancer
DPP/DPPOS: Diabetes Prevention Program/Diabetes Prevention Program Outcomes Study
DSA: Deterministic Sensitivity Analysis
EQ-5D: EuroQol 5-Dimension questionnaire
GI: Gastrointestinal
GLP-1: Glucagon-Like Peptide-1
HCC: Hepatocellular Carcinoma
HRQoL: Health-Related Quality of Life
HTA: Health Technology Assessment
ICER: Incremental Cost-Effectiveness Ratio
INMB/NMB: Incremental Net Monetary Benefit/Net Monetary Benefit
ISPOR: International Society for Pharmacoeconomics and Outcomes Research
MASLD: Metabolic Dysfunction-Associated Steatotic Liver Disease
MASH: Metabolic Dysfunction-Associated Steatohepatitis
NHS/SSR: National Health Service/Servizio Sanitario Regionale
OWSA: One-Way Sensitivity Analysis
PASSI: Progressi delle Aziende Sanitarie per la Salute in Italia (Italian national surveillance system)
PM: Project Management
PSA: Probabilistic Sensitivity Analysis
QALY: Quality-Adjusted Life Year
RCT: Randomized Controlled Trial
RR: Relative Risk
SMDM: Society for Medical Decision Making
SoC: Standard of Care
WTP: Willingness-to-Pay
