# Supplementary material for: Cost-effectiveness of an integrated lifestyle intervention for obesity in southern Italy: a model-based simulation of costs, health outcomes and gastrointestinal cancer burden
Source: Front Public Health. 2026 Oct 29;14:1956296. doi: 10.3389/fpubh.2026.1956296 (PMC13755306; doi:10.3389/fpubh.2026.1956296)
Supplement: Supplementary file 1 [file Data_Sheet_1.ZIP › S1-CHEERS-2022-checklist/CHEERS Checklist.pdf]

CHEERS 2022 Checklist

Topic	No.	Item	Location where item is reported
**Title**			
	1	Identify the study as an economic evaluation and specify the interventions being compared.	Title
**Abstract**			
	2	Provide a structured summary that highlights context, key methods, results, and alternative analyses.	Abstract
**Introduction**			
**Background and objectives**	3	Give the context for the study, the study question, and its practical relevance for decision making in policy or practice.	1. Introduction
**Methods**			
**Health economic analysis plan**	4	Indicate whether a health economic analysis plan was developed and where available.	No formal health economic analysis plan was registered prior to the analysis; the model structure, parameter set and analytic choices are reported in full in Section 2 and Supplementary Material S3.
**Study population**	5	Describe characteristics of the study population (such as age range, demographics, socioeconomic, or clinical characteristics).	2.1 Target Population
**Setting and location**	6	Provide relevant contextual information that may influence findings.	2.1 Epidemiological study design 2.2 Computational Study Design;
**Comparators**	7	Describe the interventions or strategies being compared and why chosen.	2.3 Interventions and comparators
**Perspective**	8	State the perspective(s) adopted by the study and why chosen.	2.2 Computational Study Design; 2.5 Costs, Utilities, and Model Inputs
**Time horizon**	9	State the time horizon for the study and why appropriate.	2.2 Computational Study Design; 2.4 Model overview

Topic	No.	Item	Location where item is reported
**Discount rate**	10	Report the discount rate(s) and reason chosen.	2.4 Model overview; 2.6 Sensitivity Analyses; 2.7 Worst Scenario Analysis
**Selection of outcomes**	11	Describe what outcomes were used as the measure(s) of benefit(s) and harm(s).	2.2 Study Design; 2.4 Model overview
**Measurement of outcomes**	12	Describe how outcomes used to capture benefit(s) and harm(s) were measured.	2.5 Costs, Utilities, and Model Inputs; Table 1
**Valuation of outcomes**	13	Describe the population and methods used to measure and value outcomes.	2.5 Costs, Utilities, and Model Inputs; Table 1
**Measurement and valuation of resources and costs**	14	Describe how costs were valued.	2.5 Costs, Utilities, and Model Inputs; Table 1 (micro-costing); S3
**Currency, price date, and conversion**	15	Report the dates of the estimated resource quantities and unit costs, plus the currency and year of conversion.	2.4 Model overview (constant 2024 euros; no inflation adjustment)
**Rationale and description of model**	16	If modelling is used, describe in detail and why used. Report if the model is publicly available and where it can be accessed.	2.4 Model overview; 2.10 Software Architecture and Reproducibility (repository URL); Figures 1–2
**Analytics and assumptions**	17	Describe any methods for analysing or statistically transforming data, any extrapolation methods, and approaches for validating any model used.	2.4 Model overview; 2.6 Sensitivity and Scenario Analyses; 2.8 Technical Verification; 2.9 Model validation
**Characterising heterogeneity**	18	Describe any methods used for estimating how the results of the study vary for subgroups.	No subgroup analyses were performed: the cohort is modelled as homogeneous within health states, and the model does not support stratification by age, sex or baseline severity.
**Characterising distributional effects**	19	Describe how impacts are distributed across different individuals or adjustments made to reflect priority populations.	4. Discussion (Distributional considerations)
**Characterising uncertainty**	20	Describe methods to characterise any sources of uncertainty in the analysis.	2.6 Sensitivity Analyses; 2.7 Worst Scenario Analysis

Topic	No.	Item	Location where item is reported
**Approach to engagement with patients and others affected by the study**	21	Describe any approaches to engage patients or service recipients, the general public, communities, or stakeholders (such as clinicians or payers) in the design of the study.	Patients, the public and external stakeholders were not involved in the design or conduct of this modelling study.
**Results**			
**Study parameters**	22	Report all analytic inputs (such as values, ranges, references) including uncertainty or distributional assumptions.	Table 1; S3 Model Parameters and Equations
**Summary of main results**	23	Report the mean values for the main categories of costs and outcomes of interest and summarise them in the most appropriate overall measure.	3.1 Clinical Outcomes and GI-Oncology Impact; Table 2; Table 3
**Effect of uncertainty**	24	Describe how uncertainty about analytic judgments, inputs, or projections affect findings. Report the effect of choice of discount rate and time horizon, if applicable.	3.2 Sensitivity Analyses; 3.3 Scenario Analysis
**Effect of engagement with patients and others affected by the study**	25	Report on any difference patient/service recipient, general public, community, or stakeholder involvement made to the approach or findings of the study	Patients, the public and external stakeholders were not involved in the design or conduct of this modelling study.
**Discussion**			
**Study findings, limitations, generalisability, and current knowledge**	26	Report key findings, limitations, ethical or equity considerations not captured, and how these could affect patients, policy, or practice.	4. Discussion; 5. Conclusions
**Other relevant information**			
**Source of funding**	27	Describe how the study was funded and any role of the funder in the identification, design, conduct, and reporting of the analysis	Funding

Topic	No.	Item	Location where item is reported
**Conflicts of interest**	28	Report authors conflicts of interest according to journal or International Committee of Medical Journal Editors requirements.	Conflicts of interest

From: Husereau D, Drummond M, Augustovski F, et al. Consolidated Health Economic Evaluation Reporting Standards 2022 (CHEERS 2022) Explanation and Elaboration: A Report of the ISPOR CHEERS II Good Practices Task Force. Value Health 2022;25. doi:10.1016/j.jval.2021.10.008
