# Supplementary material for: Cost-effectiveness of an integrated lifestyle intervention for obesity in southern Italy: a model-based simulation of costs, health outcomes and gastrointestinal cancer burden
Source: Front Public Health. 2026 Oct 29;14:1956296. doi: 10.3389/fpubh.2026.1956296 (PMC13755306; doi:10.3389/fpubh.2026.1956296)
Supplement: Supplementary file 1 [file Data_Sheet_1.ZIP › S2-Integrated_Obesity_Program/Supplemental_Integrated_Obesity_Program.pdf]

Integrated Obesity Management Program – Structure and Costs

Program Outline

Target: Adults with obesity
Duration: 12 months
Team: Kinesiologists, Nutritionists, General Practitioners (GPs)
Goal: Promote sustainable lifestyle change and weight loss through progressive reduction of external supervision and multidisciplinary support.

Physical Activity Pathway

Phase	Duration	Main Activities	Monthly Cost (€)	Total Cost (€)
1. Intensive (Personal Trainer)	3 months	3×/week individual supervised sessions	600	1800
2. Supervised Group	3 months	Weekly group/semi-individual sessions	100	300
3. Autonomous + Booster	6 months	Free gym access, occasional booster	50	300

- Rationale: Gradual reduction of supervision (from individual to group to autonomy), based on behavior change and self-efficacy theories (Prochaska, Bandura, Deci & Ryan).

Nutritional Pathway

Step	Duration	Main Activities	Monthly Cost (€)	Total Cost (€)
1. Individualized Start	4 months	Initial consult + monthly follow-ups	70	280
2. Group Support	4 months	Monthly group sessions, peer exchange	50	200
3. Maintenance	4 months	Group meetings, educational seminars	30	120

- Rationale: From individual advice to peer-supported group sessions, ending with educational maintenance phase. No digital tools included in this analysis.

Role of GPs

Patient selection, initial clinical evaluation, ongoing safety and integration of care.

Key Innovative Features

- Stepwise, theory-based transition from external supervision to autonomy
- Combined individual and group support
- Integration of medical, nutritional, and physical activity expertise
- Cost transparency and scalability

References

Prochaska JO, Velicer WF. The transtheoretical model of health behavior change. Am J Health Promot. 1997;12(1):38-48.

Bandura A. Self-efficacy: The exercise of control. New York: Freeman; 1997.

Deci EL, Ryan RM. Intrinsic motivation and self-determination in human behavior. New York: Plenum; 1985.

Schoeppe, S., Alley, S., Van Lippevelde, W., Bray, N. A., Williams, S. L., Duncan, M. J., & Vandelanotte, C. Efficacy of interventions that use apps to improve diet, physical activity and sedentary behaviour: a systematic review. *International Journal of Behavioral Nutrition and Physical Activity*, 18(1), 1-26. doi: 10.1186/s12966-016-0454-y.

Ashton, L. M., Morgan, P. J., Hutchesson, M. J., Rollo, M. E., & Collins, C. E. Feasibility and preliminary efficacy of the 'HEYMAN' healthy lifestyle program for young men: A pilot randomised controlled trial. *Nutrition Journal*, 21(1), 1-15.

Rodriguez-Cristobal, J. J., Alonso-Villaverde, C., Panisello, J. M., Travé-Mercade, P., Rodriguez-Cristobal, M., Serrano-Cumplido, A., ... & Barbe-Illa, F. (2020). Effectiveness of a motivational intervention on overweight/obese patients in the primary healthcare: a cluster randomized trial.
