# Supplementary material for: Cost-effectiveness of an integrated lifestyle intervention for obesity in southern Italy: a model-based simulation of costs, health outcomes and gastrointestinal cancer burden
Source: Front Public Health. 2026 Oct 29;14:1956296. doi: 10.3389/fpubh.2026.1956296 (PMC13755306; doi:10.3389/fpubh.2026.1956296)
Supplement: Supplementary file 1 [file Data_Sheet_1.ZIP › S3-Model_Parameters/Supplementary_S3_Model_Parameters.pdf]

Supplementary Material S3

Model Parameters and Probabilistic Distributions

Table S3.1 — Core model parameters

PARAMETER	MEANING	STANDARD	WORST	PSA DEVIATION STANDARD SCENARIO	PSA DEVIATION WORST SCENARIO	SOURCE / NOTES
n_patients	Size of the modelled cohort	19,000	19,000	Not sampled	Not sampled	Simulation cohort
project_years	Simulation time horizon	20	20	Not sampled	Not sampled	Simulation duration
p_success	Annual probability of achieving clinically significant weight loss (≥10% of initial body weight)	64%	44%	Beta(α = 8.36, β = 4.70)	Beta(α = 13.56, β = 17.26)	Standard: Bischoff et al., 2012 (1)(intention-to-treat analysis for 10% weight loss). Worst: Ard et al., 2019 (2)(success rate at 52 weeks in the OPTIFAST arm of the OPTIWIN trial). Both source programmes incorporate low-calorie or total meal replacement, whereas the modelled intervention comprises supervised physical activity and nutritional counselling without meal replacement. The transfer of efficacy across these delivery models is a limitation of the parameterisation; structured behavioural programmes delivered without meal replacement report smaller mean weight reductions (3,4)(Hunt et al., 2014; Ahern et al., 2017).
p_recurrence	Annual probability of relapse from the maintained weight-loss state	23%	40%	Beta(α = 19.02, β = 63.68)	Beta(α = 14.60, β = 21.90)	Standard: baseline value derived from Bischoff et al., 2012 (1). Worst: Tsai and Wadden, 2006 (5) (peak long-term weight

PARAMETER	MEANING	STANDARD	WORST	PSA DEVIATION STANDARD SCENARIO	PSA DEVIATION WORST SCENARIO	SOURCE / NOTES
						regain following very-low-calorie diets).
p_decay	Annual probability of benefit decay beyond the sustained-benefit window	1.0%	1.5%	Beta(α = 24.74, β = 2,449.26)	Beta(α = 24.61, β = 1,616.06)	Model assumption
benefit_duration	Duration of the sustained-benefit window	4 years	2 years	Discrete uniform {2, 3, 4, 5, 6} years	Discrete uniform {2, 3, 4} years	Bischoff et al., 2012 (1), whose follow-up extends to three years; the standard-scenario window of four years and the upper bound of six years are extrapolations beyond the observed period. Partial persistence of weight loss at 3.5 years is documented for a comparable behavioural programme without meal replacement (Hunt et al., 2014) (3).
p_surgery	Annual probability of transition to bariatric surgery, identical in both arms	0.37%	0.37%	Beta(α = 24.90, β = 6,705.85)	Beta(α = 24.90, β = 6,705.85)	Clinical pathways outlined by SICOB, SIE/SIO/ADI (6) and IFSO/ASMBS 2024 guidelines (7)
utility_decline (annual)	Annual background utility decline applied to all health states	−0.01	−0.01	Beta(α = 4,399.99, β = 435,599.01)	Beta(α = 4,399.99, β = 435,599.01)	Ara and Brazier, 2010 (8) general-population EQ-5D algorithm with age and age-squared terms (n = 26,679). The −0.01 annual decline is a conservative model assumption: it is steeper than the gradient implied by that algorithm across the ages spanned by the cohort (approximately −0.004 per year), and it is applied symmetrically to both arms, so it compresses

PARAMETER	MEANING	STANDARD	WORST	PSA DEVIATION STANDARD SCENARIO	PSA DEVIATION WORST SCENARIO	SOURCE / NOTES
						absolute QALYs without inflating the incremental difference.
utility_obese	Health-state utility, active obesity	0.75	0.73	Beta(α = 99.25, β = 33.08)	Beta(α = 107.27, β = 39.68)	Søltoft et al., 2009 (9) EQ-5D health-state utilities by BMI category in the general population (n = 14,416, Health Survey for England 2003).
utility_treated	Health-state utility, maintained weight loss	0.81	0.76	Derived: utility_obese + Δ ~ Gamma(k = 9.00, θ = 0.006667), cap 0.12	Derived: utility_obese + Δ ~ Gamma(k = 2.25, θ = 0.013333), cap 0.06	Søltoft et al., 2009 (9) The premium represents the difference in EQ-5D utility between the active-obesity and the reduced-weight health states, consistent with the state-transition structure of the model, rather than a gradient per unit of BMI. The direction of the effect is corroborated by Dainelli et al., 2021(10); that study measures the IWQOL-Lite, a weight-specific and non-preference-based instrument, and therefore supports the sign rather than the magnitude of the premium.
utility_surgery	Health-state utility, post-bariatric state	0.81	0.76	Derived: utility_obese + Δ ~ Gamma(k = 1.44, θ = 0.041667), cap 0.12	Derived: utility_obese + Δ ~ Gamma(k = 0.36, θ = 0.083333), cap 0.06	Xia et al., 2020 (11)
qaly_base	Baseline QALY offset applied to both arms	0	0	Not sampled	Not sampled	Baseline (no comparator)
inflation	Annual inflation rate applied over the model horizon	0%	0%	Not sampled	Not sampled	Model assumption: all costs expressed in constant euros

PARAMETER	MEANING	STANDARD	WORST	PSA DEVIATION STANDARD SCENARIO	PSA DEVIATION WORST SCENARIO	SOURCE / NOTES
discount	Annual discount rate applied to costs and QALYs	3%	6%	Not sampled	Not sampled	Drummond et al., 2015 (12)
wtp (€/QALY)	Willingness-to-pay threshold used for net monetary benefit	€30,000	€20,000	Not sampled	Not sampled	Menzel, 2021(13)
p_death (annual)	Annual all-cause mortality, identical in both arms	1%	2%	Beta(α = 5,051.01, β = 500,050.03)	Beta(α = 4,999.98, β = 244,999.02)	Global BMI Mortality Collaboration, 2016 (14); ISTAT, Indicatori demografici – Anno 2024 (15). The applied 1% parameter is a conservative approximation of the crude national all-cause mortality rate, which ISTAT reports at 11.0 per 1,000 residents.
p_dropout	Annual probability of programme drop-out	20%	30%	Beta(α = 19.80, β = 79.20)	Beta(α = 17.20, β = 40.13)	Tsai and Wadden, 2006 (5)
cost_pa1	Monthly cost of supervised physical activity, phase 1	€600	€600	Not sampled	Not sampled	Regional professional tariffs (Apulia Region)
months_pa1	Duration of physical activity phase 1	3	3	Not sampled	Not sampled	Regional professional tariffs (Apulia Region)
cost_pa2	Monthly cost of supervised physical activity, phase 2	€100	€100	Not sampled	Not sampled	Regional professional tariffs (Apulia Region)
months_pa2	Duration of physical activity phase 2	3	3	Not sampled	Not sampled	Regional professional tariffs (Apulia Region)
cost_pa3	Monthly cost of supervised physical activity, phase 3	€50	€50	Not sampled	Not sampled	Regional professional tariffs (Apulia Region)
months_pa3	Duration of physical activity phase 3	6	6	Not sampled	Not sampled	Regional professional tariffs (Apulia Region)
cost_diet1	Monthly cost of nutritional counselling, phase 1	€70	€70	Not sampled	Not sampled	Regional professional tariffs (Apulia Region)
months_diet1	Duration of nutritional counselling phase 1	4	4	Not sampled	Not sampled	Regional professional tariffs (Apulia Region)

PARAMETER	MEANING	STANDARD	WORST	PSA DEVIATION STANDARD SCENARIO	PSA DEVIATION WORST SCENARIO	SOURCE / NOTES
cost_diet2	Monthly cost of nutritional counselling, phase 2	€50	€50	Not sampled	Not sampled	Regional professional tariffs (Apulia Region)
months_diet2	Duration of nutritional counselling phase 2	4	4	Not sampled	Not sampled	Regional professional tariffs (Apulia Region)
cost_diet3	Monthly cost of nutritional counselling, phase 3	€30	€30	Not sampled	Not sampled	Regional professional tariffs (Apulia Region)
months_diet3	Duration of nutritional counselling phase 3	4	4	Not sampled	Not sampled	Regional professional tariffs (Apulia Region)
cost_dir_obese (monthly)	Monthly direct medical cost, active obesity	€146	€108	Gamma(k = 25.00, θ = 5.8400)	Gamma(k = 25.00, θ = 4.3200)	Tremmel et al., 2017 (16)
cost_indir_obese (monthly)	Monthly indirect (productivity) cost, active obesity	€75	€50	Gamma(k = 25.00, θ = 3.0000)	Gamma(k = 25.00, θ = 2.0000)	Tremmel et al., 2017 (16)
cost_dir_norm (monthly)	Monthly direct medical cost, maintained weight-loss state	€110	€86	Derived: cost_dir_obese − Δ ~ Gamma(k = 25.00, θ = 1.4400)	Derived: cost_dir_obese − Δ ~ Gamma(k = 25.00, θ = 0.8800)	Tremmel et al., 2017 (16)
cost_indir_norm (monthly)	Monthly indirect cost, maintained weight-loss state	€56	€40	Derived: cost_indir_obese − Δ ~ Gamma(k = 25.00, θ = 0.7600)	Derived: cost_indir_obese − Δ ~ Gamma(k = 25.00, θ = 0.4000)	Tremmel et al., 2017 (16)
cost_surgery	One-off cost of a bariatric procedure	€8,500	€10,000	Gamma(k = 25.00, θ = 340.0000)	Gamma(k = 25.00, θ = 400.0000)	Regional DRG tariffs (Apulia Region)
pm_cost_total	Total project-management and administrative overhead	€2,776,000	€3,800,000	Not sampled	Not sampled	Model assumption

Distribution parameters are derived from the base values by the method of moments. Dispersions are applied as relative coefficients of variation (20% for transition probabilities and cost parameters; 5% for health-state utilities; 1.4% for all-cause mortality; 1.5% for the annual utility decline), except the utility premiums, which are assigned absolute standard deviations of 0.02 (maintained weight loss) and 0.05 (post-bariatric state). These are the framework's default dispersions and were not individually calibrated to the uncertainty reported in each source study. Derived parameters are not sampled independently: each is constructed from its parent parameter through a bounded, sign-constrained increment, so that no draw can place a weight-reduced state at lower utility or higher cost than active obesity. Configuration switches not listed above are set to their neutral values in both scenarios: no-harm clamping and cumulative fading disabled, no real cost drift, project-management cost spread evenly over the horizon.

Table S3.2 — Gastrointestinal oncology module parameters

PARAMETER	MEANING	CRC	HCC	PSA DEVIATION	SOURCE / NOTES
incidence_per_100k	Baseline annual incidence per 100,000 population	82.5	21.4	Log-normal (mean-preserving), relative SD 0.20	AIOM / AIRTUM, I Numeri del Cancro in Italia, 2025. (17) National crude incidence rates for colorectal and liver cancer.
RR.Obesity	Relative risk in active obesity vs normal weight	1.30	1.80	Log-normal (mean-preserving), log-SD 0.15, floored at 1.0	CRC: Ma et al., 2013 (18) pooled RR 1.334 (95% CI 1.253–1.420) for the obese versus normal-weight BMI category, from 41 prospective studies and approximately 9,000,000 participants; rounded down to 1.30. Directionally consistent with the World Cancer Research Fund / AICR Continuous Update Project Expert Report, 2017 (19), which expresses risk per 5 kg/m² of BMI rather than by category. HCC: Sohn et al., 2021 (20) pooled HR 1.77 (95% CI 1.56–2.01) for BMI above 30 kg/m², from 28 prospective cohorts and 8,135,906 subjects; rounded to 1.80. Both source estimates are defined on colorectal and on primary liver cancer respectively; the hepatic arm is accordingly parameterised on primary liver cancer, of which hepatocellular carcinoma is the predominant histology.
RR.Treated	Residual relative risk in maintained weight loss vs normal weight	1.15	1.40	Derived: 1 + (RR.Obesity − 1) × (1 − effect_weightloss); bounded within [1, RR.Obesity]	Derived quantity, not an independent input
effect_weightloss	Fraction of obesity-attributable excess risk reversed by maintained weight loss	0.50	0.50	Beta(α = 191.58, β = 191.58), 95% CI half-width ±0.05, capped at 0.80	Model assumption. Direct evidence, *predominantly from bariatric-surgery cohorts,* quantifying the reversibility of adiposity-attributable cancer risk after non-surgical weight loss is not available; the resulting uncertainty is propagated through the PSA rather than assumed away. For context, a systematic literature review of intentional weight loss and associated cancer incidence reports

PARAMETER	MEANING	CRC	HCC	PSA DEVIATION	SOURCE / NOTES
					colorectal cancer risk reductions ranging from 20% to 60% across the observational studies identified, an interval within which the assumed value of 0.50 falls; the same review records that the one randomised trial of intensive lifestyle intervention found no significant association between intentional weight loss and cancer risk (21).
cost_year1	First-year cost of an incident case	€22,000	€25,000	Log-normal (mean-preserving), log-SD 0.20	CRC: Francisci et al., 2013 (22) "initial phase" hospital costs in Italy (€13,351). The study reports expenditure incurred in 2006 in the Tuscany and Veneto registry areas and was published in 2013; the value was uplifted to the 2024 analysis price year. HCC: A four-centre Italian survey of intermediate and advanced hepatocellular carcinoma reports direct healthcare costs per patient of €26,106 for transarterial radioembolisation and €13,419 for transarterial chemoembolisation (2013–2014 values) (23).The order of magnitude is corroborated by Varela et al., 2026 (24) mean direct medical cost of first-year active management in the Spanish National Health System (€26,426).
cost_followup	Annual follow-up cost of a prevalent case	€3,700	€5,000	Log-normal (mean-preserving), log-SD 0.25	CRC: Francisci et al., 2013 (22) "continuing phase" costs in Italy (€2,238, expenditure incurred in 2006), uplifted to the 2024 analysis price year. HCC: Varela et al., 2026 (24) annual cost of surveillance and recurrence management following curative-intent treatment.
du_year1	First-year utility decrement of an incident case	0.28	0.35	Logit-normal, relative SD 0.30	CRC: Ness et al., 1999 (25) utility valuations for colorectal cancer outcome states; the 0.28 acute decrement sits within the measured range (0.26 for Stage I/II, 0.33 for Stage III). HCC: model assumption, anchored on Ishinuki et al.,

PARAMETER	MEANING	CRC	HCC	PSA DEVIATION	SOURCE / NOTES
					2022 (26) for the existence and the direction of the decrement. That meta-analysis reports a pooled EQ-5D utility of 0.8127 for hepatocellular carcinoma against 0.8413 for healthy subjects, a contrast that reflects prevalent disease rather than the acute treatment phase; the 0.35 first-year decrement applied here is therefore an assumption for the acute phase, for which stage-specific preference-based valuations were not available.
du_followup	Annual follow-up utility decrement	0.15	0.20	Logit-normal, relative SD 0.30	CRC: Ness et al., 1999 (25) utility valuations for post-treatment and remission states; the 0.15 decrement reflects the chronic rather than the acute phase. HCC: model assumption for the chronic phase, anchored on Ishinuki et al., 2022 (26) as above.
latency_years	Latency between sustained weight loss and change in incidence	3	3	Not sampled	Model assumption reflecting the physiological lag between sustained weight loss and a measurable reduction in carcinogenesis risk. Prevents overestimation of immediate oncological benefit in the early years of the simulation.
stream_l	Length of the cost and utility kernel applied to each avoided case	5 years	5 years	Not sampled	Model assumption: the costs and utility decrements attributable to an avoided case are distributed over a five-year kernel from the year of avoidance, rather than accruing entirely in a single year.
stream_alpha	Per-year continuation factor of the follow-up stream	0.23	0.23	Not sampled	Model assumption: the follow-up component of the kernel is attenuated year on year, giving a cumulative follow-up multiplier of 0.30 rather than the full five-year duration. Conservative with respect to the oncological cost offsets.
incidence_trend_pct	Annual trend in baseline incidence	0%	0%	Not sampled	Model assumption: baseline incidence held constant over the horizon, in the absence

PARAMETER	MEANING	CRC	HCC	PSA DEVIATION	SOURCE / NOTES
					of validated projections for the modelled population.
effect_half_life_years	Half-life of the risk-reversal effect	0	0	Not sampled	Model assumption: the risk-reversal effect is held constant for as long as weight loss is maintained, with no additional waning beyond relapse and benefit decay.
discount_rate	Annual discount rate applied within the module	3% / 6%	3% / 6%	Not sampled — follows the scenario	As per the main model

Values are identical in the Standard and Worst Scenario; only the discount rate follows the scenario. Dispersions for log-normally distributed parameters are expressed as the standard deviation of the logarithm, not as coefficients of variation; all log-normal draws are mean-preserving. The log-SD of 0.15 applied to the two relative risks is wider than the confidence intervals reported in the corresponding source meta-analyses (which imply approximately 0.03 for colorectal and 0.065 for liver cancer), and is therefore conservative with respect to parameter uncertainty. Baseline module inputs are also specified in the gi_params block of the configuration files accompanying this Supplementary Material.

References

1. Bischoff SC, Damms-Machado A, Betz C, others. Multicenter evaluation of an interdisciplinary 52-week weight loss program for obesity with regard to body weight, comorbidities and quality of life - a prospective study. *International Journal of Obesity* (2012) 36:614–624. doi: 10.1038/ijo.2011.107

2. Ard JD, Lewis KH, Rothberg A, others. Effectiveness of a Total Meal Replacement Program (OPTIFAST Program) on Weight Loss: Results from the OPTIWIN Study. *Obesity* (2019) 27:22–29. doi: 10.1002/oby.22303

3. Hunt K, Wyke S, Gray CM, Anderson AS, Brady A, Bunn C, Donnan PT, Fenwick E, Grieve E, Leishman J, et al. A gender-sensitised weight loss and healthy living programme for overweight and obese men delivered by Scottish Premier League football clubs (FFIT): a pragmatic randomised controlled trial. *The Lancet* (2014) 383:1211–1221. doi: 10.1016/S0140-6736(13)62420-4

4. Ahern AL, Wheeler GM, Aveyard P, Boyland EJ, Halford JCG, Mander AP, Woolston J, Thomson AM, Tsiountsioura M, Cole D, et al. Extended and standard duration weight-loss programme referrals for adults in primary care (WRAP): a randomised controlled trial. *The Lancet* (2017) 389:2214–2225. doi: 10.1016/S0140-6736(17)30647-5

5. Tsai AG, Wadden TA. The evolution of very-low-calorie diets: an update and meta-analysis. *Obesity (Silver Spring)* (2006) 14:1283–1293. doi: 10.1038/oby.2006.146

6. SICOB, SIE/SIO/ADI, IFSO/ASMBS. Italian Guidelines for the Management of Severe Obesity. (2024).

7. Eisenberg D, Shikora S, Aarts E, al et. 2022 American Society for Metabolic and Bariatric Surgery (ASMBS) and International Federation for the Surgery of Obesity and Metabolic Disorders (IFSO): Indications for Metabolic and Bariatric Surgery. *Surgery for Obesity and Related Diseases* (2022) 18:1345–1356. doi: 10.1016/j.soard.2022.08.013

8. Ara R, Brazier JE. Populating an Economic Model with Health State Utility Values: Moving toward Better Practice. *Value in Health* (2010) 13:509–518. doi: 10.1111/j.1524-4733.2010.00700.x

9. Søltoft F, Hammer M, Kragh N. The association of body mass index and health-related quality of life in the general population: data from the 2003 Health Survey of England. *Qual Life Res* (2009) 18:1293. doi: 10.1007/s11136-009-9541-8

10. Dainelli L, Luo DR, Cohen SS, others. Health-Related Quality of Life in Weight Loss Interventions: Results from the OPTIWIN Trial. *International Journal of Environmental Research and Public Health* (2021) 18:1785. doi: 10.3390/ijerph18041785

11. Xia Q, Campbell JA, Ahmad H, Si L, de Graaff B, Otahal P, Palmer AJ. Health state utilities for economic evaluation of bariatric surgery: A comprehensive systematic review and meta-analysis. *Obesity Reviews* (2020) 21:e13028. doi: 10.1111/obr.13028

12. Drummond MF, Sculpher MJ, Claxton K, Stoddart GL, Torrance GW. *Methods for the Economic Evaluation of Health Care Programmes*. 4th ed. Oxford University Press. (2015).

13. Menzel PT. How Should Willingness-to-Pay Values of Quality-Adjusted Life-Years Be Updated, and According to Whom? *AMA Journal of Ethics* (2021) 23:E601-606. doi: 10.1001/amajethics.2021.601

14. Di Angelantonio E, Bhupathiraju SN, Wormser D, Gao P, Kaptoge S, De Gonzalez AB, Cairns BJ, Huxley R, Jackson CL, Joshy G, et al. Body-mass index and all-cause mortality: individual-participant-data meta-analysis of 239 prospective studies in four continents. *The Lancet* (2016) 388:776–786. doi: 10.1016/S0140-6736(16)30175-1

15. ISTAT. Indicatori demografici – Anno 2024. Rome, Italy: Istituto Nazionale di Statistica. (2025). https://www.istat.it/comunicato-stampa/indicatori-demografici-anno-2024/ [Accessed August 1, 2026]

16. Tremmel M, Gerdtham UG, Nilsson PM, Saha S. Economic burden of obesity: a systematic literature review. *International Journal of Environmental Research and Public Health* (2017) 14:435. doi: 10.3390/ijerph14040435

17. Associazione Italiana di Oncologia Medica (AIOM), AIRTUM, Fondazione AIOM, others. *I Numeri del Cancro in Italia 2025*. Roma: Intermedia Editore. (2025).

18. Ma Y, Yang Y, Wang F, Zhang P, Shi C, Zou Y, Qin H. Obesity and Risk of Colorectal Cancer: A Systematic Review of Prospective Studies. *PLoS ONE* (2013) 8:e53916. doi: 10.1371/journal.pone.0053916

19. World Cancer Research Fund, American Institute for Cancer Research. Diet, Nutrition, Physical Activity and Colorectal Cancer. Continuous Update Project Report. (2017).

20. Sohn W, Lee HW, Lee S, Lim JH, Lee MW, Park CH, Yoon SK. Obesity and the risk of primary liver cancer: A systematic review and meta-analysis. *Clin Mol Hepatol* (2021) 27:157–174. doi: 10.3350/cmh.2020.0176

21. Liao C, Schapiro D, Mojdami D, Sheffield KM, Hoog MM, Keni R, Gathirua-Mwangi WG, Kan H. Intentional Weight Loss and Associated Cancer Incidence Among People With Overweight or Obesity: A Systematic Literature Review. *Endocrino Diabet & Metabol* (2025) 8:e70104. doi: 10.1002/edm2.70104

22. Francisci S, Guzzinati S, Mezzetti M, others. Cost profiles of colorectal cancer patients in Italy based on individual patterns of care. *BMC Cancer* (2013) 13:329. doi: 10.1186/1471-2407-13-329

23. Colombo G, Cammà C, Attili A, Ganga R, Gaeta G, Franzini JM, Volpe M, Turchetti G, Brancaccio G. Patterns of treatment and costs of intermediate and advanced hepatocellular carcinoma management in four Italian centers. *TCRM* (2015)1603. doi: 10.2147/TCRM.S88208

24. Varela M, Feliu J, Garrido-Siles M, Gómez-Bravo M, González S, Irurzun J, L Ruiz De Alda, Vieitez P, Carcedo D, Crespo M, et al. Economic burden of recurrence in patients with hepatocellular carcinoma after curative-intent treatment in Spain. *Health Econ Rev* (2026) doi: 10.1186/s13561-026-00796-y

25. Ness RM, Holmes AM, Klein R, Dittus R. Utility Valuations for Outcome States of Colorectal Cancer. *American Journal of Gastroenterology* (1999) 94:1650–1657. doi: 10.1111/j.1572-0241.1999.01157.x

26. Ishinuki T, Ota S, Harada K, Kawamoto M, Meguro M, Kutomi G, Tatsumi H, Harada K, Miyanishi K, Kato T, et al. Current standard values of health utility scores for evaluating cost-effectiveness in liver disease: A meta-analysis. *WJG* (2022) 28:4442–4455. doi: 10.3748/wjg.v28.i31.4442
